# AOX1-Subfamily Gene Members in *Olea europaea* cv. “Galega Vulgar”—Gene Characterization and Expression of Transcripts during IBA-Induced in Vitro Adventitious Rooting

**DOI:** 10.3390/ijms19020597

**Published:** 2018-02-17

**Authors:** Isabel Velada, Dariusz Grzebelus, Diana Lousa, Cláudio M. Soares, Elisete Santos Macedo, Augusto Peixe, Birgit Arnholdt-Schmitt, Hélia G. Cardoso

**Affiliations:** 1 Departamento de Fitotecnia, ICAAM—Instituto de Ciências Agrárias e Ambientais Mediterrânicas, Universidade de Évora, Pólo da Mitra, Ap. 94, 7006-554 Évora, Portugal; ivelada@uevora.pt (I.V.); apeixe@uevora.pt (A.P.); 2 Institute of Plant Biology and Biotechnology, Faculty of Biotechnology and Horticulture, University of Agriculture in Kraków, 31-120 Kraków, Poland; d.grzebelus@ogr.ur.krakow.pl; 3 ITQB NOVA, Instituto de Tecnologia Química e Biológica António Xavier, Universidade Nova de Lisboa, Av. da República, 2780-157 Oeiras, Portugal; dlousa@itqb.unl.pt (D.L.); claudio@itqb.unl.pt (C.M.S.); 4 Functional Cell Reprogramming and Organism Plasticity (FunCrop), EU Marie Curie Chair, ICAAM, Universidade de Évora, 7006-554 Évora, Portugal; elisetemcd@gmail.com; 5 Functional Genomics and Bioinformatics, Department of Biochemistry and Molecular Biology, Federal University of Ceará, 60020-181Fortaleza, Brazil; 6 Science and Technology Park Alentejo (PACT), 7005-841 Évora, Portugal

**Keywords:** vegetative propagation, olive, adventitious rooting, auxins, IBA, plant mitochondria, alternative oxidase, alternative polyadenylation, transposable elements, gene expression

## Abstract

Propagation of some *Olea europaea* L. cultivars is strongly limited due to recalcitrant behavior in adventitious root formation by semi-hardwood cuttings. One example is the cultivar ”Galega vulgar”. The formation of adventitious roots is considered a morphological response to stress. Alternative oxidase (AOX) is the terminal oxidase of the alternative pathway of the plant mitochondrial electron transport chain. This enzyme is well known to be induced in response to several biotic and abiotic stress situations. This work aimed to characterize the alternative oxidase 1 (AOX1)-subfamily in olive and to analyze the expression of transcripts during the indole-3-butyric acid (IBA)-induced in vitro adventitious rooting (AR) process. *OeAOX1a* (acc. no. MF410318) and *OeAOX1d* (acc. no. MF410319) were identified, as well as different transcript variants for both genes which resulted from alternative polyadenylation events. A correlation between transcript accumulation of both *OeAOX1a* and *OeAOX1d* transcripts and the three distinct phases (induction, initiation, and expression) of the AR process in olive was observed. Olive *AOX1* genes seem to be associated with the induction and development of adventitious roots in IBA-treated explants. A better understanding of the molecular mechanisms underlying the stimulus needed for the induction of adventitious roots may help to develop more targeted and effective rooting induction protocols in order to improve the rooting ability of difficult-to-root cultivars.

## 1. Introduction

Olive (*Olea europaea* L.) is one of the oldest agricultural fruit crops worldwide and is mostly cultivated for olive oil production. Olive orchards are predominantly concentrated in the Mediterranean basin [1], although they have recently expanded to new regions due to the importance of olive oil in the human diet. Portugal has a production area of 430,000 ha of olive orchards, which represents about 5% of the world olive oil production. Portuguese olive oils are known worldwide for their exceptional organoleptic characteristics. Nowadays, olive plants are mostly propagated by semi-hardwood cuttings, a process in which adventitious root formation is a key factor. However, some of the agronomically interesting Portuguese olive cultivars used for oil production have been revealed to be recalcitrant to adventitious rooting (AR), which leads to a reduced availability of those varieties in the nurseries that are to be used in new orchard plantations. For example, ”Galega vulgar” usually presents average rooting rates of 5–20% when semi-hardwood cuttings are used, being considered a difficult-to-root cultivar [2]. Similar recalcitrant behaviour has been described for autochthone cultivars with high agronomical interest in different countries (for review see [3]). In this frame, the study of AR in *O. europaea*, in view of the optimization of the process in stem cuttings of recalcitrant olive cultivars, has become an important research topic, which requires fundamental and applied research at different levels.

The process of AR at the base of stem cuttings is considered a morphological response to stress [4] that can be influenced by a large number of interacting internal and external factors. It involves hormone-transmitted metabolic changes, molecular transduction pathway activation, protein degradation, and protein de novo synthesis, as well as adaptive global genome regulation (for review see [3]). The cutting’s removal from the mother tree and subsequent treatment with auxin are both stress factors that are highly involved in cell response towards AR. AR, as a directed growth response, can be supposed as a plant strategy to diminish stress exposure [5].

Mitochondria, as a physical platform for networks, signal perception, and signal canalization, play a central role in plant cell response to fluctuating cellular conditions, such as is often seen in environmental stresses, and in the further reacquisition of metabolic homeostasis [6,7,8]. The alternative respiratory pathway, localized in mitochondria, has been a relevant research topic regarding plant stress acclimation and adaptation in many reports focused on the involvement of the alternative oxidase (AOX; EC 1.10.3.11 ubiquinol:O_2_ oxidoreductase id IPR002680) gene family members [6,7,8,9,10,11]. Clifton et al. [12,13] pointed to the importance of this alternative respiratory pathway as an early-sensing system for cell programming. The involvement of AOX in AR has been also taken as an important research topic, not only in view of understanding the role of the genes during the AR process, but also to further develop functional markers in order to be able to select genotypes that show efficient cell reprogramming [4,14,15].

AOX is a terminal quinol oxidase located on the matrix side of the inner mitochondrial membrane, and it works as the key enzyme in the alternative respiratory pathway. It has been described in a wide variety of species from different kingdoms, like plants, protists, fungi, and also in some animals [16]. However, AOX has been best studied in the plant kingdom, particularly in angiosperm plant species [17,18,19], where it is often encoded by a small multigene family composed of one to six genes distributed in two discrete gene subfamilies termed *AOX1* and *AOX2* [7,17,19,20,21]. The number of *AOX* genes and their distribution within the two subfamilies is species-specific [19]. Due to the high diversity in terms of gene duplication pattern [17], some *AOX* classification schemes for angiosperm plant species have been developed [17,22,23]. In dicot plant species, genes belonging to both subfamilies have been described. Only recently, the *AOX2*-subfamily was identified in species within the *Araceae* family [18], which is due to the availability of increasing information regarding monocot whole genome sequencing data.

AOX can play a number of roles in the optimization of the respiratory metabolism and in the integration of the respiratory metabolism with other metabolic pathways that impact the supply of or demand for carbon skeletons, reducing power and ATP [6,10,11]. This enzyme also modulates the levels of signalling molecules, thus supporting the crosstalk between the metabolic status of mitochondria and the nucleus that regulates gene expression [8]. For a long time, genes belonging to the *AOX1*-subfamily have been implicated in plant responses to a diversity of abiotic and biotic stresses (for reviews see [7,8,24]), while *AOX2*-subfamily members were described as housekeeping genes or more involved in plant development [13,25,26]. Nevertheless, the paradigm that *AOX1*-subfamily members are the only ones related to the stress response has been challenged [12,25,26,27].

In the context of AR in olive, the *AOX2*-subfamily gene member has been the main focus of AOX research. In addition to understanding the involvement of *AOX* gene members in the AR process, and in view of the development of further functional markers that are able to discriminate between genotypes with different potential to develop adventitious roots, gene sequence variability has been investigated [4,14,15]. *AOX* sequence variability, located in the protein coding and non-coding regions, has been reported in different plant species [4,28,29,30,31,32]. However, despite the studies carried out by direct mutagenesis to investigate the effect that a specific single nucleotide polymorphism (SNP) has on the protein functionality (see overview in [33]), there are few reports in natural systems showing the link between sequence polymorphisms and changes in the phenotype. Abe et al. [34] were the first research group to indicate the relevance of AOX polymorphisms in abiotic stress tolerance by identifying in the *Oryza sativa AOX1a* a SNP that mapped to a region of a QTL for low temperature tolerance in anthers at the booting stage. More recently, Hedayati et al. [15] reported the existence of two SNPs located at intron 3 of *OeAOX2* that correlate with differences in rooting ability.

In addition to the SNPs present within *AOX* gene sequences, other forms of sequence variability have been reported, whose differential processing can be influenced by physiological conditions, such as cell growth, differentiation, development, or pathological events [35]. Variability at the 3′-UTR sequence that encompasses sequence and length variability due to alternative polyadenylation (APA) events has been reported in *AOX* gene members from different plant species [4,36]. APA allows a single gene to encode multiple mRNA transcripts. Depending on the location of the alternative poly(a) signal (PAS), APA events may affect gene expression qualitatively by the production of different protein isoforms, or quantitatively if miRNAs binding sites and/or other regulatory elements are concerned that can act as negative regulators.

Considering that stress stimulus is a key factor for the AR process to be successful, it becomes relevant to investigate the involvement of the *AOX1*-subfamily members in the process, as well as to explore whether mechanisms related to the gene expression regulation might be involved in AR. Here, we characterize the gene members of the *AOX1* sub-family at the cDNA and gDNA levels, and we analyze the expression of its transcripts during AR in olive.

## 2. Results

### 2.1. Characterization of AOX1-Subfamily Members

**Figure 1:**
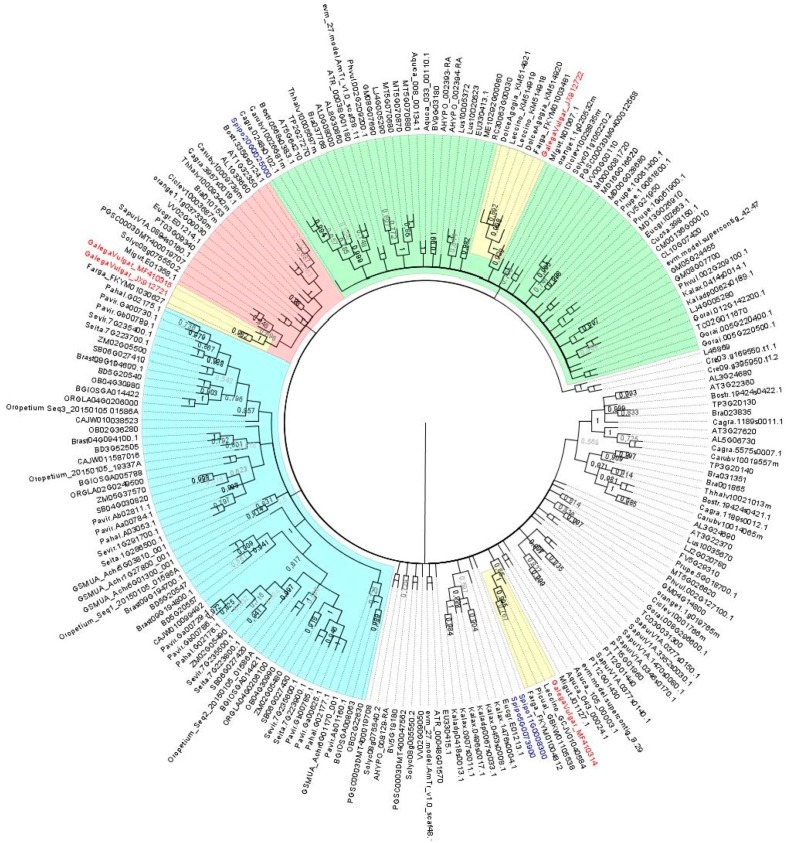
Neighbor-Joining (NJ) tree showing the relationships among deduced AOX sequences from 56 plant species, including monocot and eudicot plant species. Putative peptide sequences corresponding to the isolated *AOX1*-subfamily members of *Olea europaea* L. were included (shown in red). 206 AOX sequences from higher plants were included (correspondence of accession numbers and the plant species is included in supplementary Tables S3 and S4). The NJ tree was obtained using the complete peptide sequences. The alignments were bootstrapped with 1000 replicates by the NJ method using the MEGA 7 software. AOX sequence from *Neurospora crassa* and two sequences of *Chlamydomonas reinhardtii* were used as outgroups. The scale bar indicates the relative amount of change along branches. In green: the branch corresponding to the AOX2-subfamily members. AOX1d members are in red and AOX1 members from monocot plant species are in the branch colored in light blue. Clusters grouping olive AOX members are in yellow and accessions corresponding to AOX from cv. ”Galega vulgar” are in red (*OeAOX1a*, acc. no. MF410314; *OeAOX1d*, acc. no. MF410315 and JX912721; OeAOX2, acc. no. JX912722).

In a first attempt to clarify the information about the composition of the *AOX1*-subfamily in olive, a blast search was carried out at the web page of the olive whole genome sequencing project that uses the *O. europaea* L. cv. ”Farga” as target genome (Oe6 browser at http://denovo.cnag.cat/genomes/olive/). For that search, *AOX1a* sequence from *Arabidopsis thaliana* L. deposited at the NCBI GenBank (acc. no. AT3G22370) was used. Three sequences with high similarity were identified. A blast search made at the wgs NCBI database also identified those sequences, corresponding to the scaffolds Oe6_s00216c09 (acc. no. FKYM01004812.1), Oe6_s05781c34 (acc. no. FKYM01030627.1), and Oe6_s00133c14 (acc. no. FKYM01003481.1). Additionally, a blast search using the same *AtAOX1a* sequence was made at the NCBI databases nr/nt and transcriptome shotgun assembly (TSA), which allowed for the identification of complete *OeAOX* sequences from cv. ”Leccino” (acc. no. GCJV01040584, KM514918, and KM514919), cv. “Picual” (acc. no. GBKW01105538), and cv. “Dolce Agogia” (acc. no. KM514920 and KM514921). Extracted sequences were used to construct a dendrogram using the in silico translated sequences together with sequences retrieved from 56 plant species at Phytozome and Plaza databases, which include monocot and eudicot plant species. To determine the relationship between the OeAOX from *O. europaea* and those retrieved sequences, a NJ tree was constructed using the translated sequences. There are clearly two different clusters composed by OeAOX1-subfamily members and a single cluster corresponding to the OeAOX2-subfamily members from all *O. europaea* cultivars (in yellow the three clusters that include OeAOX sequences) (Figure 1). Members from cv. “Galega vulgar” belonging to the AOX1 clusters were named as OeAOX1a (acc. no. MF410314) and OeAOX1d (acc. no. MF410315 and JX912721), the later one considering the high similarity with the AOX1d members from different plant species, and the AOX2 as OeAOX2 (acc. no. JX912722). A clear separation of AOX2-subfamily members can be seen (cluster in green). AOX1 members from monocot plant species form a separated group within the AOX1-subfamily (cluster in light blue). *AOX1*-subfamily members identified from other olive cultivars, not annotated as *AOX* gene members, appeared deposited as TSA in both cases transcribed upon cold stress.

**Figure 2:**
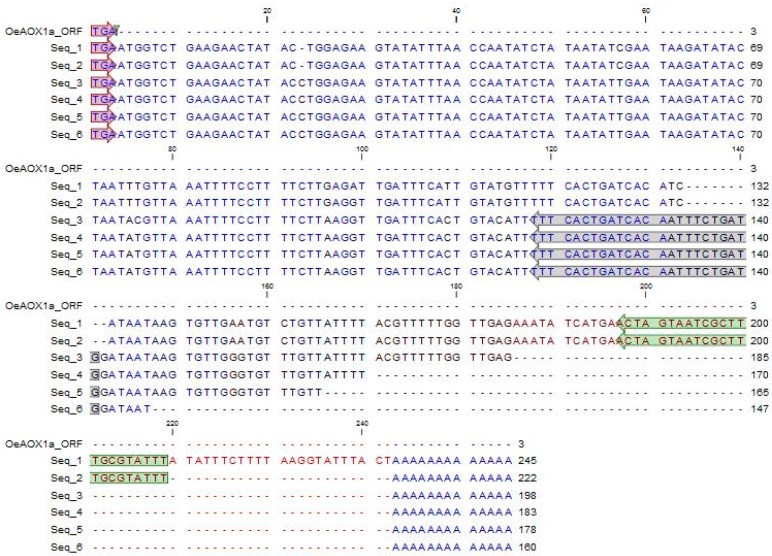
Alignment of the six different isolated sequences corresponding to the 3′-UTR of *OeAOX1a* gene. The sequences are presented starting at the stop codon TGA shown in red. The reverse primers used in RT-qPCR analysis for each transcript variant are shown in different colors (green: variant X1, acc. no. MF410314; grey: variant X2, acc. no. MG208095) (for primers sequence see Table S2).

Based on the available information [14], it was possible to successfully produce the 5′ and 3′ ends of both *AOX1*-subfamily members in cv. ”Galega vulgar”. RACE work developed for the isolation of 3′ ends of both *OeAOX1* gene members allowed the identification of high sequence variability due to different APA events. *OeAOX1a* transcripts presented the coding region unchanged but the 3′-UTR length variable, which ranged between 144 and 229 bp (Figure 2). Figures S1 and S2 show the complete cDNA sequences of *OeAOX1a*_transcript variant X1 with 1462 bp (deposited at the NCBI as acc. no. MF410314) and *OeAOX1a*_transcript variant X2 with 1249 bp (acc. no. MG208095). *OeAOX1a* sequences present an open reading frame (ORF) of 1086 bp, which encodes a putative polypeptide of 362 amino acid residues, that corresponds to a putative peptide with 40.6 kDa and a pI of 8.19.

Variability at the 3′ end of *OeAOX1d* was not due to 3′-UTR length size variability but to differences on the protein coding sequence, also due to an APA event. This event gave rise to two different transcripts named as variant X1 and variant X2 (deposited at the NCBI with the acc. no. MF410315 and JX912721, respectively) with 1277 bp (Figure S3) and 1597 bp (Figure S4), respectively. While *OeAOX1d*_transcript variant X1 results from the transcription of the four exons, typical of the general structure of plant *AOX* composed by four exons interrupted by three introns, transcript variant X2 includes the transcription of the N-terminal region of intron 3 with transcript cleavage located 125 bp downstream the 5′ conserved GT dinucleotide at splice site (described below). This sequence variability can lead to different putative peptide sequences. Transcript variant X1 is characterized by an ORF with 996 bp, which encodes a polypeptide with 332 aa and the variant X2 with an ORF with 1062 bp that encodes a polypeptide with 354 aa, which will give a putative peptide with 38 (pI of 6.7) and 40.7 kDa (pI 7.79), respectively.

**Figure 3:**
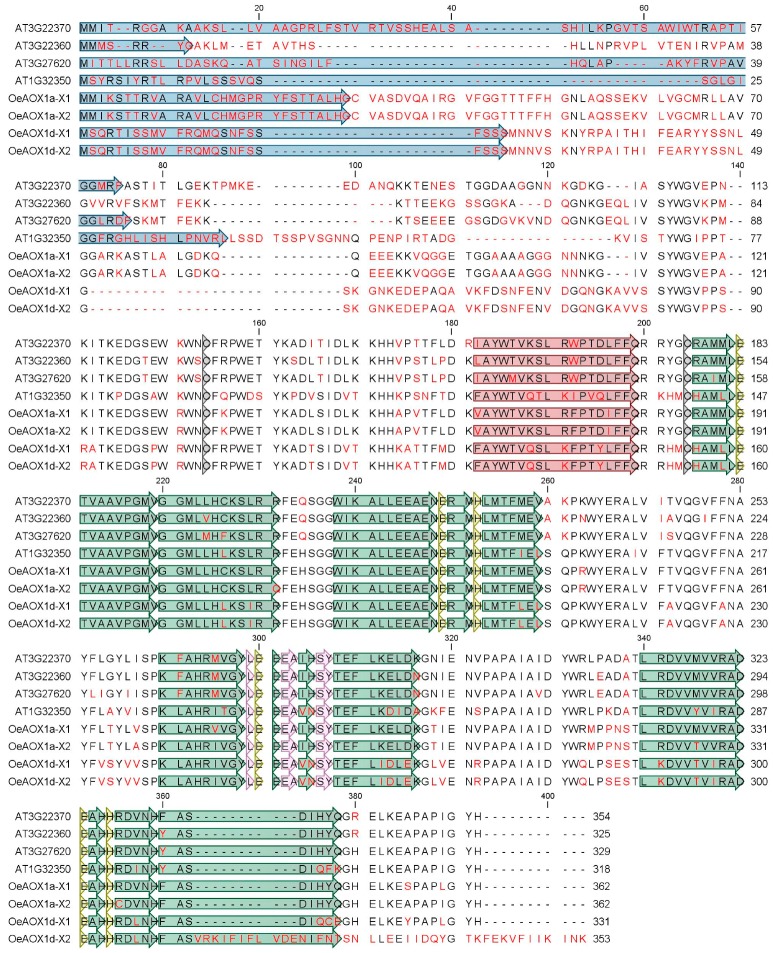
Multiple alignment of putative amino acid translated sequences of previously reported AOX proteins from *A. thaliana* (*AtAOX1a*_AT3G22370, *AtAOX1b*_AT3G22360, *AtAOX1c*_AT3G27620, *AtAOX1d*_AT1G32350) and AOX *from O. europaea* L. cv. ”Galega vulgar” (*OeAOX1a*_transcript variant X1_MF410314 and *OeAOX1a*_transcript variant X2_MG208095, *OeAOX1d*_transcript variant X1_ MF410315 and *OeAOX1d*_ transcript variant X2_JX91272). The alignment was performed using CLC Main Workbench 6.7.1 software. The data were retrieved from public web-based database Plaza v2.5, freely available at http://bioinformatics.psb.ugent.be/plaza/versions/plaza_v2_5/. Amino acid residues differing are shown in red, deletions are shown by minus signs. The putative mitochondrial transit peptides (mTP) are shown in blue boxes. The sites of two conserved cysteins (CysI and CysII) involved in dimerization of the AOX protein by S–S bond formation [37] are indicated in dark grey boxes. Helices α1 and α4, which form the hydrophobic region on the AOX molecular surface and are involved in membrane binding, are shown in red; helices α2, α3, α5, and α6, which form the four-helix bundle accommodating the diiron center, are shown in green [38]. Amino acids residues that coordinate the diiron center (E, glutamate and H, histidine) and those that interact with the inhibitor are in yellow and light pink boxes, respectively.

Figures S1–S4 indicate the cDNA sequences for *AOX1* genes including the putative translated peptide and the conserved sites for intron positions. The difference in the overall length for the complete ORF sequences between *OeAOX1a* and transcript variant X1 of *OeAOX1d* (considering for this last one the transcript with the conserved gene structure) is due to the size variability at the N-terminal region of exon 1. The first exon has a size of 411 bp for *OeAOX1a* and 318 bp for *OeAOX1d*_transcript variant X1. Variation on both *OeAOX1d* complete ORF sequences is due to the exon 4, which shows 57 bp in transcript variant X1, leading to a peptide sequence homologous to the most common sequence across higher plants (see polypeptide alignment in Figure 3).

Forward and reverse gene specific primers located at the 5′ and 3′ gene ends, respectively, were used at the genomic level and allowed the isolation of both *OeAOX1* gene members: *OeAOX1a* with 2215 bp and *OeAOX1d* with 2054 bp length (from start to stop codon). To identify gene structure, genomic and transcript sequences were used at the Splign software. A four exons structure showing size conservation at the three last exons (exon 2: 129, exon 3: 489 and exon 4: 57 bp), interrupted by three size variable introns, was identified at the *OeAOX1a* and *OeAOX1d* (Figures S5 and S6). In both *OeAOX1* genes, introns were flanked by a GT sequence at the 5′ end and an AG at the 3′ end known as donor and acceptor splicing sites, respectively.

Comparing the isolated sequences from cv. ”Galega vulgar” with the sequences available at the whole genome databases from cv. ”Farga”, high conservation was found at the protein encoding sequence. However, high variability was found at intonic regions (see Figure S7). In silico analysis also revealed that *OeAOX1d*_transcript variant X2 cannot be transcribed on cv.”Farga” due to the existence of a stop codon previous to the polyadenylation site (also known as the poly(A) site—PAS) [39].

A search for the identification of putative sequences coding for miRNAs located at the intronic regions revealed their absence in both *OeAOX1a* and *OeAOX1d*. Also, no other regulatory elements related to transposable elements and repetitive sequences were identified within gene sequences of cv. ”Galega vulgar”. In *OeAOX1d* genomic sequence of cv. ”Farga”, a putative transposable element (data not shown) was identified. The availability of upstream and downstream sequences of both *OeAOX1* genes in the cv. ”Farga” allowed us to search for transposable elements located in the vicinity of both genes and to perform the analysis of promoter region to scan for *cis*-elements regulated by auxins. The in silico analysis allowed the identification of several copies of copia and gypsy LTR retrotransposons. Details of the identified full length LTR insertions are presented in Table 1. Directly upstream to *OeAOX1a*, there were two gypsy elements; a copy of 84856_A was nested within 95401_A (Figure S8). The latter element was inserted ca. 1 Kb upstream from the start codon of *OeAOX1a*. Interestingly, another copy of 95401_A was found directly upstream from the *OeAOX1d* gene (Figure S9), albeit at a larger distance (ca. 3.5 Kb) to the start codon and in the opposite orientation as related to the copy associated with *OeAOX1a*. Repetitive sequences were present directly downstream *OeAOX1d*; however, owing to the lack of contiguous assembly of that region, a detailed analysis was not performed.

*OeAOX1a* and *OeAOX1d* promoter sequences up to 1.5 kbp upstream from the translation start site were scanned using PlantCARE and New Place software’s for the identification of auxin *cis*-acting regulatory elements (CAREs). Four CAREs were identified in the *OeAOX1a*’s promoter region and 3 CAREs were identified in the *OeAOX1d* (see details in Table S5). From the four CAREs identified in *OeAOX1a*, three were located at a region prior to −500 bp upstream of the translation start site (New PLACE IDs: NTBBF1ARROLB, ARFAT and SURECOREATSULTR11). In the case of *OeAOX1d*, from the three motifs identified, only one is located closer to the start codon, prior to −500 bp upstream of the translation start site (New PLACE ID: ASF1MOTIFCAM, with sequence TGACG, at position −164). Comparing CAREs of the promoter regions for both *OeAOX1* genes, only one is common, the New PLACE ID: NTBBF1ARROLB, with sequence ACTTTA. However, in terms of its location, the sequence ACTTTA is not conserved between the two genes. It is located at −266 upstream from the translation start site for *OeAOX1a*, while for *OeAOX1d*, it is located at −668 and repeated at +1109. In addition, it is interesting to note that the sequence of auxin response factor binding site found in promoters of primary/early auxin responsive genes (New PLACE ID: ARFAT, with sequence TGTCTC) was only identified in *OeAOX1a* (at positions +305 and −306).

A multiple sequence alignment including AOX1-translated peptides from *A. thaliana* and *O. europaea* cv. ”Galega vulgar”was used to highlight similarities and differences in the putative protein sequences (Figure 3). OeAOX1a and OeAOX1d encoded by both transcript variants (X1 and X2) revealed structural features usually found in most of the higher plants’ AOX with the identification of two conserved cysteines (CystI and CystII) and di-iron-binding sites. Sequence diversity at the C-terminal region is here restricted to putative peptide of *OeAOX1d*_transcript variant X2. This change is implicated in the sequence of helice α6, one of the four-helix bundles accommodating the diiron center.

**Figure 4:**
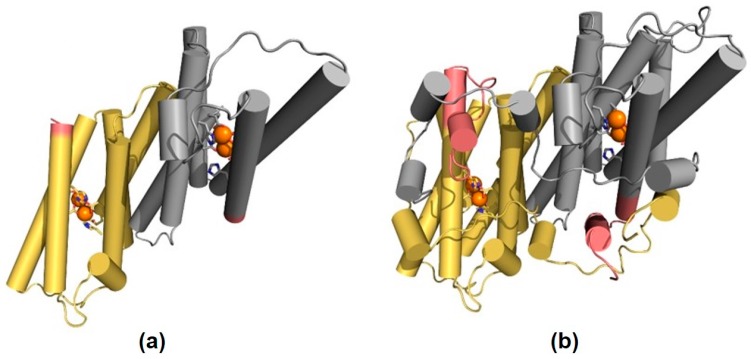
Structural mapping of the sequence diversity of OeAOX1d. The homology-based model of (**a**) OeAOX1d_transcript variant X2 and the structure of (**b**) AOX from *T. brucei* are displayed using a cartoon representation, with the helices shown as cylinders. The two identical functional subunits are colored in yellow (subunit A) and grey (subunit B), and the iron atoms that form the diiron center are represented by orange spheres, with the coordinating residues displayed using sticks. The region that corresponds to the sequence change in OeAOX1d_transcript variant X2 is highlighted in pink.

In order to gain insight into the possible protein structural effects of the sequence change present in *OeAOX1d*_transcript variant X2, we turned to the structure of the AOX from *Trypanosoma brucei* (PDB ID: 3VV9) [38], which is the only homologous protein whose structure is available. Unfortunately, the N- and C-terminal regions of OeAOX1 do not align well with the sequence of AOX from *T. brucei* and, therefore, we could only model the region between helices α2 and α6 (Figure 4A). However, based on the alignment of OeAOX1d and AOX from *T. brucei*, we can infer that the sequence change of *OeAOX1d*_transcript variant X2 is located between the end of helix α6 (one of the four-helix bundle accommodating the diiron center) and the C-terminus (Figure 4B). The region that is affected by this change is close to the diiron center and is also implicated in inter-subunit interactions in the dimeric form of AOX.

High sequence diversity was detected at the N-terminal region, which consequently lead to high diversity on the mitochondrial transit peptide (see Figure 3). Both putative OeAOX1 translated peptides were predicted to be localized in mitochondria (mTP score of 0.672 and 0.604 regarding the OeAOX1a and OeAOX1d, respectively). The predicted length of the cleavage site of the mitochondrial targeting sequence for OeAOX1a and OeAOX1d is 28 and 45 amino acid residues, respectively. The predicted mitochondrial transit peptide is shown in the alignment of Figure 3, in which no conservation across protein sequences is visible, not within the AOX1-subfamily members across species and not even within the AOX1-subfamily genes from a single plant species.

### 2.2. Analysis of Transcript Expression

#### 2.2.1. *OeAOX1* Genes are Differentially Expressed during IBA-induced AR

**Figure 5:**
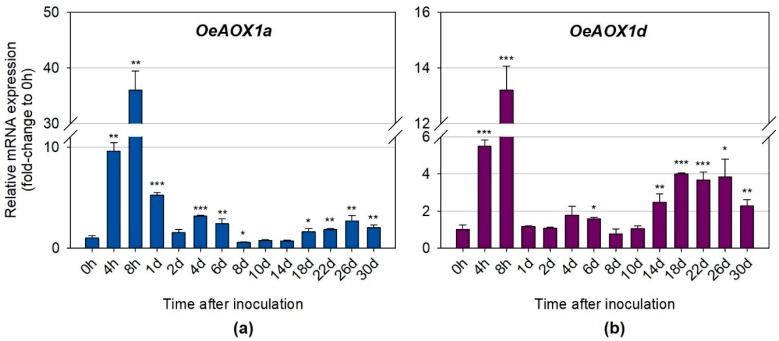
Relative mRNA expression of (**a**) *OeAOX1a* and (**b**) *OeAOX1d* in stem basal segments of *O. europaea* L. microcuttings during IBA-induced adventitious rooting. *OeACT* and *OeEF1a* were used as reference genes in data normalization. The relative expression values are depicted as the mean ± standard deviation of four biological replicates for each time point. The bars represent the fold-change related to the time point 0 hours after microcuttings treatment and inoculation, which was set to 1. Statistical significances (* *p* ≤ 0.05, ** *p* ≤ 0.01, and *** *p* ≤ 0.001) between the two means were determined by the *t*-test using IBM^®^ SPSS^®^ Statistics version 22.0 (SPSS Inc., Armonk, NY, USA), h: hours, d: days.

In order to verify whether the expression levels of *OeAOX1a* and *OeAOX1d* genes were changed during IBA-induced rooting, quantitative real time PCR was performed. Both *OeAOX1a* and *OeAOX1d* genes showed a similar expression pattern throughout the rooting assay (Figure 5A,B). The maximum peak of up-regulation for both genes occurred at 8 h after IBA treatment. *OeAOX1a* showed, however, higher levels of expression at this time point (36.0-fold change, *p* ≤ 0.01) (Figure 5A) than *OeAOX1d* (13.2-fold change, *p* ≤ 0.001) (Figure 5B) when compared to the levels observed at the corresponding controls (0 h, without IBA treatment). Looking at days 1 and 2, which can be seen as the recovery time point after the maximum peak of expression, it can be observed that both genes decreased drastically reaching even, at day 2, expression values close to the ones observed at 0 h. A second increment of expression for both genes, although lower than the first increment, was observed at day 4. Here, and again, the expression levels at this time point, and compared with the ones observed at 0 h, were higher for *OeAOX1a* (3.2-fold change, *p* ≤ 0.001) than for *OeAOX1d* (1.8-fold change, not statistically significant). Day 4 corresponds to the end of the induction phase and beginning of the initiation phase in AR process in olive [40]. From this time point forward, the expression levels decreased again reaching the minimum peak at day 8, for both genes. A third increment occurs for both genes around days 10–14. Around days 12–14 corresponds to the time when calli formation becomes apparent before root emergence. From these time points on and until the end of the rooting trial (30 days), and in opposition to what was previously observed, the expression levels for *OAOX1a* were lower than the ones observed for *OeAX1d*. For example, at day 22, which corresponds to the end of the initiation phase and the beginning of the expression phase of the AR process [40], *OeAOX1a* had a slight 1.8-fold (*p* ≤ 0.01) increase, whereas *OeAOX1d* had a higher increment of 3.7-fold (*p* ≤ 0.001) when compared to the expression levels of the corresponding controls.

#### 2.2.2. Distinct Transcripts Variants Show Different Expression Levels

**Figure 6:**
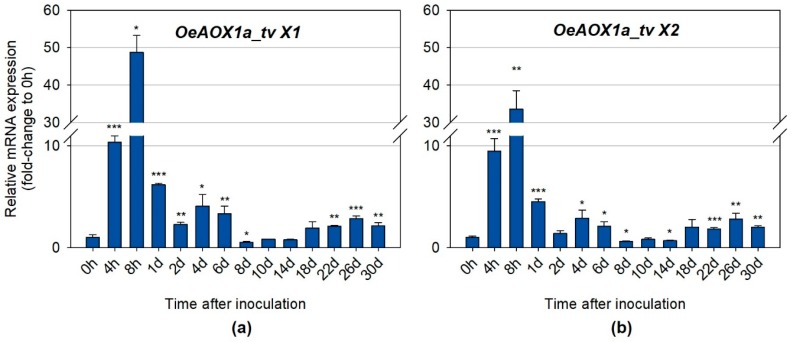

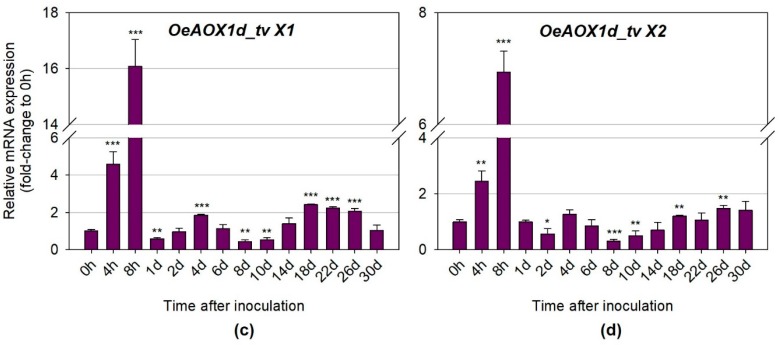
Relative mRNA expression of (**a**) *OeAOX1a*_transcript variant X1, (**b**) *OeAOX1a*_transcript variant X2, (**c**) *OeAOX1d*_transcript variant X1, and (**d**) *OeAOX1d*_transcript variant X2 (D) in stem basal segments of *O. europaea* L. microcuttings during IBA-induced adventitious rooting. *OeACT* and *OeEF1a* were used as reference genes in data normalization. The relative expression values are depicted as the mean ± standard deviation of four biological replicates for each time point. The bars represent the fold-change related to the time point 0 hours after stem microcuttings treatment and inoculation, which was set to 1. Statistical significances (* *p* ≤ 0.05, ** *p* ≤ 0.01 and *** *p* ≤ 0.001) between the two means were determined by the *t*-test using IBM^®^ SPSS^®^ Statistics version 22.0 (SPSS Inc., Armonk, NY, USA), h: hours, d: days, tv: transcript variant.

Quantitative real time PCR was also performed to further investigate whether the distinct transcript variants (primers were designed for a specific region of each transcript variant) for each gene (*OeAOX1a* and *OeAOX1d*), produced due to APA events, were differentially expressed during IBA-induced AR. *OeAOX1a* transcripts with longer 3′-UTRs (*OeAOX1a*_transcript variant X1) (Figure 6A) showed higher expression levels at the time points corresponding to the first and second increments than the transcripts with shorter 3′-UTRs (*OeAOX1a*_transcript variant X2) (Figure 6B). For example, the expression levels at 8 h in relation to the control (time point 0 h) for *OeAOX1a*_transcript variant X1 were about 48.7–fold higher (*p* ≤ 0.05), while for *OeAOX1a*_transcript variant X2 was around 33.5-fold higher (*p* ≤ 0.01). At day 4, variant X1 showed a 4.1-fold change (*p* ≤ 0.05) and variant X2 a 2.9-fold change (*p* ≤ 0.05) compared with the time point 0 h. A very similar expression pattern throughout the rooting assay was observed for both transcript variants. On the contrary, the shorter *OeAOX1d* transcripts composed by the four exons (*OeAOX1d*_transcript variant X1) (Figure 6C) showed higher expression levels at these time points (8 h: 16.1-fold change, *p* ≤ 0.001; 4 days: 1.8-fold change, *p* ≤ 0.001) than the longer transcripts with an alternative PAS located at the intron 3 (*OeAOX1d*_transcript variant X2) (Figure 6D) and lacking the exon 4 sequence (8 h: 6.9-fold change, *p* ≤ 0.001; 4 days: 1.3-fold change, not statistically significant). As for *OeAOX1a*, a similar expression profile over all the time points tested was observed for both variants. Additionally, the expression profile of transcript variants was very similar to the expression profile exhibited by the *OeAOX1a* and *OeAOX1d* genes (including all sets of transcripts, since primers were designed in a common region). From day 14 onwards, which corresponds to the time point when roots start to emerge, both *OeAOX1a* transcript variants (with shorter (variant X2) and longer (variant X1) 3′-UTRs) showed a similar level of expression between them when compared to the levels of the corresponding controls. On the other hand, *OeAOX1d* transcripts corresponding to the variant X1 were more expressed than the variant X2. While the expression levels of *OeAOX1a* gene correlate to the ones shown by each transcript variant, in the case of *OeAOX1d* gene this does not happen. The expression levels of *OeAOX1d* gene were higher (almost double) than the expression levels of the most expressed transcript variant (variant X1). This result suggests that *OeAOX1d* gene may have other transcript variants that were not analysed here separately, despite the fact that they have been detected by the primers designed to a common region among the transcript variants.

## 3. Discussion

Correct classification of homologous *AOX* genes across plant species is challenging [32]. However, it gains high importance when the physiological role of those genes should be comparable across species. Thus, classification is a dynamic process that needs regular updates to develop knowledge [23]. Based on the most recent classification system available for *AOX*, basal angiosperms and eudicots contain both *AOX*-subfamilies (*AOX1* and *AOX2*) subdivided into specific types (*AOX1a*-*c*/*e*, *AOX1d*, *AOX2a-c,* and *AOX2d*) [17]. Here, 206 sequences from public data bases were analysed. The distribution of the putative translated peptide encoded by the isolated *OeAOX1* sequences in two different clusters within the main cluster of AOX1-subfamily revealed the existence of two *AOX1*-subfamily members in olive. One of those sequences clustered together with the *AOX1d* member of *A. thaliana* (AT1G32350) and the sequence of *Solanum lycopersium* available at the NCBI as *AOX1b* (NP_001234120.1) but renamed by Costa et al. [17] as *AOX1d*. Considering this homology, the olive member was named as *OeAOX1d* and submitted to the NCBI databases at cDNA and gDNA levels (acc. no. MF410315, JX912721, and MF410319). Putative AOX2 translated peptides from different *O. europaea* cultivars clustered together within the AOX2-subfamily, confirming a single *AOX2* member in this species.

Across kingdoms, there is a lack of a general pattern with respect to intron/exon structure in *AOX* genes [16]. However, within plants, the most common gene structure described for *AOX* comprises four exons interrupted by three introns [41,42]. Exceptions to this gene structure have been reported in some *AOX* gene members and different plant species, due to events of intron loss and gain [19]. From the known examples, an intron loss leads to a structure of three exons, and an intron gain to a structure of five-exons. Despite the typical structure of four exons, *AOX* gene members are well known by exons size conservation achieved at the three last exons (129, 489 and 57 bp, respectively). The combination between gene homology, gene structure, and exons size allows us to be more confident about the classification of a gene as a member of the *AOX* family. Cases of miss-annotation regarding *AOX* gene members and genes from another membrane-bound di-iron carboxylate protein, the plastid terminal oxidase (PTOX; EC 1.10.3.11 ubiquinol:O_2_ oxidoreductase id IPR002680), are still common, since *AOX* and *PTOX* share high level of homology [32]. In general, the exon size conservation in the 4-exons structure of *AOX* gene members is one of the factors that contributes to the low variability in protein size. Normally, exon 1 is the one that is variable not only in length but in sequence composition as well. Protein size typically ranges between 32–41 kDa depending on the species [22,43,44]. Putative translated OeAOX1 peptides from cv. “Galega vulgar” showed, by in silico analysis, a size that is in range from 38–40.7 kDa. Both OeAOX1a and OeAOX1d were predicted, with high score, to be located in the mitochondria. However, high variability in the N-terminal region was observed within AOX1 members. Sequence variability located in that gene region was previously reported across AOX members within and between species [31,41,42]. Nevertheless, how this variability can affect the regulation of gene expression and/or the protein transport and activity is still not known. The N-terminal region determines interaction with the protein transport system that regulates integration into the organelle. In many cases, amino acids comprising the signal peptide are cleaved off the protein once they reach their final destination. A comparison between *A. thaliana* and *O. sativa* using a high set of proteins showed high variability at that region, going from 19 to 109 amino acids in *A. thaliana*, and from 18 to 117 amino acids in *O. sativa* [45]. Specifically on AOX, Campos et al. [41] described mTP sequence length variability across plant species and between protein isoforms within the same plant species. More recently, a study that aimed the identification of allelic variation within the *AOX1* gene member considering 39 carrot genotypes described high variability at that region, going from a mTP sequence with 20 to 41 amino acid residues [31].

Contrarily to the conservation at AOX protein coding sequences, high variability can be seen in protein non-coding regions, which include introns and untranslated regions (5′-UTR and 3′-UTR) [4,15,28,29,30,31,32,42]. Size and sequence variability located at these regions can have an important physiological role. Gene architecture, which considers not only the number but also the length of exons and introns, is nowadays considered as one important regulatory player. Several studies indicate that gene architecture toward short genes with few introns allows for efficient expression during short cell cycles. In contrast, genes composed by long introns can be expected to exhibit delayed expression [46]. Long introns are described as a timing mechanism that works for biological signal feedback regulatory networks [47]. Despite this role in regulation of gene expression, which is associated with gene expression delays, it is also known that the presence of introns could enhance gene transcription [48]. Some introns harbour non-coding RNAs (e.g., miRNAs and snoRNAs) for which the processing from introns can speed up or slow down the rate of expression of the host gene [49]. Despite the fact that no miRNAs were identified within the *OeAOX1* sequences, an in silico analysis, performed to search for TEs, revealed the existence of several putative retrotransposons located in the adjacent regions upstream and downstream of the AOX gene position. TEs located in intergenic regions (up- or downstream target genes) or within a gene sequence (promoter or intron sequences) may provide regulatory elements affecting gene expression through a variety of mechanisms (for review see [50]). In plants, TEs can constitute from ca. 10% of *A. thaliana* genome (Arabidopsis Genome Initiative 2000) to 85% of the B73 *Zea mays* genome [51]. Several reports point out the existence of TEs within *AOX* gene sequences [52,53,54]. These results lead us to hypothesize that expression of *AOX* genes might be influenced by adjacent TEs.

Simultaneously, introns offer the potential for regulatory functions such as alternative splicing and APA events. It is nowadays evident that APA acts as a major mechanism of gene regulation being widespread across all eukaryotic species [39]. In plants, it was reported that 70% of *A. thaliana* genes and around 50% of *O. sativa* genes have at least one alternative poly(A) site [55]. In some specific cases, variability in transcripts is associated with regulation of flowering time, growth, and developmental processes [56,57]. As a result of APA events, a single pre-mRNA can produce more than one mRNA. If alternative PAS is located in internal introns/exons, APA events may lead to the production of different protein isoforms with differences in subcellular localization, stability, or function by changing or completely removing functional domains (for review see [39]). It may also result in unstable mRNA isoforms with a negative feedback on gene expression by generation of truncated transcripts that are recognized and degraded by a specific mechanism named nonsense-mediated decay (NMD) [58]. From *OeAOX1d*, two putative protein isoforms could be produced due to an alternative PAS located at intron 3: (a) *OeAOX1d*_transcript variant X1 with four exons that encodes the structural feature typical of AOX in plants, and (b) *OeAOX1d*_transcript variant X2 with three exons and a partial sequence of intron 2 that replaced exon 4. This latter transcript encodes a putative truncated protein that lacks 19 amino acids at the C-terminal end. When this transcript variant was analyzed during IBA-induced rooting assay, it demonstrated lower differential expression levels (compared to the control) than variant X1. The sequence alteration present in the *OeAOX1d*_transcript variant X2 will affect the structure of helix α6, which can have an impact on the coordination of the diiron center. This can be predicted by referring to the structure of AOX from *T. brucei* [38]. In AOX from *T. brucei*, the C-terminal region is involved in important inter-subunit interactions. Therefore, it is possible that the sequence alteration present in the *OeAOX1d*_transcript variant X2 will also affect interaction between the two polypeptide chains. This can have implications on the stability of the dimer. However, further studies will be required to investigate whether both transcripts would be translated to two different protein isoforms, and if so, whether both are functional. Additionally, if PAS are located in the 3′-UTRs, APA events will lead to the synthesis of transcripts conserving unchanged the protein coding sequence but presenting different 3′-UTR lengths. 3′-UTR length can affect the transcript stability, localization, transport, and translational properties [35]. 3′-UTRs often harbor miRNAs binding sites and/or other regulatory elements [59] that can act as negative regulators, mostly of larger transcripts. Many mRNAs use 3′-UTR alternative PAS to achieve tissue-specific expression and function [60,61]. We found in our study alternative PAS located at the 3′-UTR of the *OeAOX1a,* which generates short and long 3′-UTRs. The identification of *OeAOX1a* sequences carrying 3′-UTR regions with different sizes may suggest the possibility of differential post-transcriptional regulation. It should be noted that in our system (IBA-induced AR in olive explants), although both *OeAOX1a* transcript variants X1 and X2 (carrying 3′-UTR with different sizes) showed a similar expression pattern throughout the process, indicating co-regulation, *OeAOX1a*_transcript variant X1 showed more pronounced differential expression levels (compared to the control) than variant X2, up to day 8. Differential 3′-UTR sizes of a gene can have positive, negative, or even neutral effects on mRNA stability and on the resulting protein levels depending on whether the availability of RNA-binding sites, such as miRNA-binding sites, is influenced [62,63,64,65]. Many different RNA-binding proteins (RBPs) and a variety of signals located at that transcript region can regulate mRNA localization, decay, and translation [65].

The expression of both genes, *OeAOX1a* and *OeAOX1d*, was dramatically increased in the early stages of rooting with the maximum peak of transcript accumulation at 8 h after IBA treatment. Three previous studies addressed the involvement of *AOX* genes in the process of IBA-induced AR [4,14,15]. They were based on the earlier raised hypothesis, which proposed *AOX* as a functional marker candidate for efficient adventitious rooting of *O. europaea* L. [66,67]. Santos Macedo et al. [4,14] showed first in semi-hardwood shoot cuttings of an easy-to-root olive cultivar (cv. ”Cobrançosa”) that root induction was significantly reduced by treatment with an inhibitor of AOX activity (salicylhydroxamic acid—SHAM). This observation could be confirmed in an in vitro system for olive propagation (cv. ”Galega vulgar”), thus pointing to the general importance of *AOX* genes during the process of induced rooting [14]. In the latter work, it could be shown that SHAM-inhibition was in fact specific to rooting and did not interfere with preceding callus formation. This observation was later confirmed by Porfirio et al. [68] using the same experimental system. In a first approach, *OeAOX2* was identified as a promising gene candidate for functional marker development towards improving rooting efficiency in olive [4]. First evidence of an association between rooting ability and *OeAOX2* gene expression in olive cuttings was then provided by Hedayati et al. [15]. This group also confirmed the presence of polymorphisms in *OeAOX2* with a possible correlation to distinct rooting behavior. The present work adds new information to the rooting system of olive by showing the expression of the *OeAOX1a* and *OeAOX1d* genes by quantitative real time PCR during IBA-induced AR in microcuttings.

Adventitious rooting is considered a developmental process organized in a sequence of interdependent stages [69,70,71,72,73,74]. It includes three phases: (1) induction, corresponding to the period preceding any visible histological occurrence, with molecular and biochemical events; (2) initiation, starting with the first histological events, like root primordia organization; here, small cells with large nuclei and dense cytoplasm start to be apparent; and (3) expression, involving the development of the typical dome shape structures, intra-stem growth, and emergence of root primordial [75,76,77,78]. In olive, induction phase corresponds to the first 4 days after microcuttings treatment and inoculation, when cells regain meristematic features. From 4 until 14 days, the first meristemoids and morphogenetic root zones were observed, events corresponding to the initiation phase. These events are followed by high mitotic activity that eventually leads to the expression phase, which starts at 22 days after the root-inducing treatment [40]. From our results, it can be seen that both *OeAOX1a* and *OeAOX1d* genes exhibited three increments in their expressions throughout the rooting assay. The first one, as mentioned above, was the most pronounced, and occurred at 8 h after microcuttings treatment and inoculation, and corresponded to the beginning of the induction phase. The second increment was observed at 4 days, which corresponds to the end of the induction phase and beginning of the initiation phase. The third increment was observed from the end (14 days for *OeAOX1d* and 18 days for *OeAOX1a*) of the initiation phase onwards. It is likely that the first observed increment may be related to the stress associated with the cut injury and auxin treatment. In fact, as suggested by Santos Macedo et al. [4], the initial cut of olive microcuttings and its subsequent treatment with auxins may constitute a stress to the involved cells, and therefore olive AR can be seen as stress-induced reprogramming of shoot cells [4,66,67]. The second and third increments may be related to the role that AOX might have on cell differentiation and growth/development. The link between AOX and cell differentiation and plant growth has been indicated by several reports [7,67,79,80].

Interestingly, Porfirio et al. [68], using the same experimental system, observed elevated free IAA (indole-3-acetic acid) levels in the first hours after treatment, peaking also at 8 h. It would be worth investigating further this correlation between the levels of free IAA observed by Porfirio et al. [68] and the accumulation of *AOX1* transcripts found in the present study during the induction phase of olive rooting. It is likely that a link may exist between altered auxin homeostasis and induced *OeAOX* gene expression, as suggested by others [81,82], during AR in olive. In this context, it would be desirable to investigate also genes involved in the auxin signaling and transport.

It is tempting to speculate that, in our study, *AOX* genes were highly induced, probably by increased levels of ROS as a consequence of a stressful situation (cut injury plus auxin treatment) and by IBA application to promote rooting. Indeed, it has been reported that different abiotic stresses are likely to cause the formation of different ROS signatures in plant cells (for review see [83]). Moreover, the production of ROS by mitochondria was suggested to be a critical factor for the induction of AOX [13,84], which in turn regulates the amounts of ROS, and therefore AOX has a large impact on redox regulation at a cellular level on environmental stresses [7]. Auxins can induce the production of ROS [85] and regulate ROS homeostasis [86], hinting at the relationship between auxin signaling and oxidative stress [85]. Thus, the marked accumulation of *OeAOX* transcripts at 8 h observed in our study may also be the result of the increased levels of free IAA detected by Porfírio et al. [68], which possibly contributed to elevate the levels of ROS. Moreover, in silico analysis at the *OeAOX1* promoter region in search of cis-acting regulation elements identified different auxin responsive elements (AuxREs) that could be involved in regulation of *AOX* gene expression. The identification of different AuxREs in the promoter region of both *OeAOX1* genes allows us to speculate that regulation might be done in a differential way. Presence of the TGTCTC-motif in *OeAOX1a*, which belongs to the family of Auxin Response Factors (ARFs) (see review in [87]), could explain the higher increase in gene expression in comparison to *OeAOX1d* that lacks this motif.

In summary, two genes were identified as members of the *AOX1*-subfamily in the cv.”Galega vulgar”, with both showing the most common structure of *AOX* gene members with four exons interrupted by three introns. Alternative polyadenylation (APA) events were responsible for the production of transcript variants in both genes. *OeAOX1a* transcript variants show different 3′-UTR lengths with no changes at the protein coding sequence. *OeAOX1d* shows an alternative PAS located at the intron 3 that leads to the synthesis of one transcript variant showing a truncated protein coding sequence that lacks the exon 4 sequence. The sequence alteration found in the *OeAOX1d*_transcript variant X2 will prevent the structure of helix α6 having an impact on the coordination of the diiron center and also it can affect the interaction between the two polypeptide chains having implications in the stability of the dimer. Our findings showed a strong correlation between *OeAOX1a* and *OeAOX1d* transcripts accumulation and the three distinct phases (induction, initiation, and expression) of the AR process in olive, with the expression of these genes more pronounced at the induction phase. The elevated IBA-induced expressions at this phase may be related to the stressful conditions associated with AR process and the application of IBA for rooting induction. A possible link between OeAOX1 induction and altered auxin homeostasis in olive AR may exist, since *OeAOX1* transcripts were increased at the same time point for which earlier studies showed elevated levels of free auxins. Additionally, different transcript variants for each gene studied here, although showing a similar expression pattern, demonstrated different levels of expression during AR, which would be worth exploring further. Further studies will be required to clarify whether the diverse transcripts encountered may give rise to distinct functional protein isoforms and also to understand the physiological role of such variability. Taken together, these results contribute to a better understanding of the molecular mechanisms underlying the stress stimulus needed for the induction of adventitious roots. Thus, the results may allow us to develop more targeted and effective rooting induction protocols, which in turn can help to increase the rooting ability of difficult-to-root cultivars.

## 4. Materials and Methods

### 4.1. Characterization of the AOX Genes at the cDNA and Genomic Levels

#### 4.1.1. Plant Material

*Olea europaea* L. explants of cv. ”Galega vulgar” (clone 1441), established under in vitro conditions since 2005, were maintained until today following the procedure described by Peixe et al. [88]. The derived in vitro grown plantlets were used for gene isolation. Leaves were collected from a single plantlet and used for total RNA and genomic DNA (gDNA) extractions.

#### 4.1.2. Isolation of Complete *OeAOX1*-Subfamily Gene Members

The isolation of complete gene sequences was performed in several steps. The first one was based on the protocol described by Saisho et al. [21] for isolation of the *AOX* gene members in *A. thaliana* and further referred to by different authors for gene isolation in different plant species [41,42,79]. The isolation of mainly two different sequences belonging to the *AOX1*-subfamily was previously reported by Santos Macedo et al. [14]. Based on that information, gene specific primers were designed in order to isolate gene ends of the identified *OeAOX* gene fragments by 5′ and 3′ RACE-PCRs. Total RNA used for cDNA synthesis was previously extracted using the RNeasy Plant Mini Kit (Qiagen, Hilden, Germany) with on-column digestion of DNA applying the RNase-Free DNase Set (Qiagen, Hilden, Germany), according to the manufacturer’s protocol. The concentration of total RNA and its purity was determined with the NanoDrop-2000C spectrophotometer (Thermo Scientific, Wilmington, DE, USA). For both ends, 1 μg of total RNA was used to synthesize the first-strand cDNA using the SMARTerTM RACE cDNA Amplification kit (Clontech Laboratories, Inc., Mountain View, CA, USA) according to the manufacturer’s instructions. RACE-PCRs were carried out separately using 1 μL of the corresponding single strand cDNA as template and 0.2 μM of the forward/reverse gene specific primers (depending if 3′ or 5′ end isolation) (Table S1) combined with 0.2 μM of the Universal Primer Mix (provided with the kit) following the instructions recommended by the manufacturer. PCRs were all carried out in a 2770 thermocycler (Applied Biosystems, Foster City, CA, USA).

For complete gene isolation, gDNA was isolated using the DNeasy Plant Mini Kit (Qiagen, Hilden, Germany) according to the manufacturer’s protocol. The amount of gDNA and its purity was determined with the NanoDrop-2000C spectrophotometer (Thermo Scientific, Wilmington, DE, USA). One gene-specific primer set was designed for each *OeAOX* gene (Table S1) based on the previously isolated 5′ and 3′-UTR sequences. Ten ng of gDNA were used as template with 0.2 μM of each specific primers. PCRs were performed using the Phusion™ High-Fidelity DNA Polymerase (Finnzymes, Espoo, Finland) according to the manufacturer’s protocol. PCR was carried out in a 2770 thermocycler (Applied Biosystems, Foster City, CA, USA) running for 35 cycles each one consisting in 10 s at 98 °C, 30 s at 55 °C, and 2 min at 72 °C. An initial step at 98 °C for 30 s and a final step at 72 °C for 10 min were used.

#### 4.1.3. Cloning and in Silico Sequence Analysis

PCR fragments were separately cloned into a pGem^®^-T Easy vector (Promega, Madison, WI, USA) and used to transform *E. coli* JM109 (Promega, Madison, WI, USA) competent cells. Plasmid DNA was further extracted from putative recombinant clones by using the GeneJET Plasmid Miniprep kit (Thermo Scientific, Vilnius, Lithuania) and further sequenced in sense and antisense strands (Macrogen company, Seoul, South Korea: www.macrogen.com).

CLC Main Workbench 7.5.1 software (ClCbio, Aarhus N, Denmark) was used to edit sequences. Intron location was made using the software Splign (https://www.ncbi.nlm.nih.gov/sutils/splign/splign.cgi?textpage=online&level=form).

In order to clarify the question related to the number of genes that compose the *AOX1*-subfamily, a blast search using the *AOX1* from *A. thaliana* L. (acc. no.AT3G22370) deposited at the NCBI—National Center for Biotechnology Information (GenBank) was made at the web page of olive genome databases (http://denovo.cnag.cat/genomes/olive/) using the Oe6 browser. To get confirmation, the retrieved sequences were then blasted at the NCBI data bases using the BLAST algorithm [89] (http://www.ncbi.nlm.nih.gov/) (BLASTn) at the whole-genome shot gun contigs (wgs). To identify *AOX* sequences from other olive cultivars, a BLASTn search using the same sequence was made at different NCBI databases (nucleotide collection, nr/nt; transcriptome shotgun assembly, TSA; expressed sequence tags, est).

To perform a comparison between AOX proteins from higher plants, sequences were retrieved from the whole genomes available at the Plaza (http://bioinformatics.psb.ugent.be/plaza/) and the Phytozome (https://phytozome.jgi.doe.gov/pz/portal.html) databases using a BLAST search analysis based on the exon 3 as the most conservative region across *AOX* genes and plant species.

Sequences retrieved were aligned in MUSCLE (http://www.ebi.ac.uk/Tools/msa/muscle/) following the standard parameters. Phylogenetic reconstruction was performed in MEGA 7 software [90] by Neighbor-Joining (NJ) and the inferred tree was tested by bootstrap analysis using 1000 replicates, “number of differences” as the substitution model, and “pairwise deletion” for gaps/missing data treatment. Graphical view was edited in the Fig Tree v14.0 software (http://tree.bio.ed.ac.uk/software/figtree/).

The freely available TargetP software [91] was used to predict the protein subcellular localization and the position of the cleavage sites of mitochondrial targeting signals (http://www.cbs.dtu.dk/services/TargetP/) using the translated peptide corresponding to exon 1. The prediction of putative isoelectric point (pI) and the molecular weight was obtained using the freely available tool PeptideMass at the Expasy software (http://web.expasy.org/peptide_mass/).

#### 4.1.4. Homology-Based Model

The model of AOX1d was generated using the structure of AOX from *T. brucei* (PDB ID: 3VV9) [38] as a template. Only the region between residues 159 and 313 was modelled, since the N- and C-terminal regions did not align well with the template. The model was built using the software modeler [92], version 9.6, and setting the refinement degree to slow. The final model corresponds to the one with the lowest value of the objective function, out of 20 generated structures.

#### 4.1.5. In Silico Identification of Regulatory Elements Located at the *AOX* Gene Boundaries

For the identification of putative miRNA precursor sequences located at the introns and UTRs of the isolated *OeAOX* genes, the publicly available software miR-abela (http://www.mirz.unibas.ch/cgi/pred_miRNA_genes.cgi) was used. Further steps related with pre-miRNAs validation, prediction of the secondary structure of predicted pre-miRNA, screening of potential miRNAs, and identification of target genes candidates were developed according to the procedure described by Velada et al. [42].

For identification of transposable elements (TE) in the vicinity of the *OeAOX* genes (using olive genome sequencing information), contigs encompassing these genes were self-aligned using Blast2Seq at the NCBI (blast.ncbi.nlm.nih.gov). The contigs were also used as queries to search GeneBank nucleotide database restricted for the *Olea* genus (taxid:4145). Hits matching known *O. europaea* TEs were retained. Regions flanked by putative LTRs not showing similarity to known *Olea* retrotransposons were used as queries for Censor (www.girinst.org) to search for the most similar elements in the RepBase. Target site duplications (TSDs) were identified manually. Additional manipulations and alignments were performed in BioEdit 7.2.5 [93]. All names of olive LTR retrotransposons are reported after Barghini et al. [94].

For identification of transposable elements (TE) in the vicinity of the *OeAOX* genes (using olive genome sequencing information), contigs encompassing these genes were self-aligned using Blast2Seq at the NCBI (blast.ncbi.nlm.nih.gov). The contigs were also used as queries to search GeneBank nucleotide database restricted for the *Olea* genus (taxid:4145). Hits matching known *O. europaea* TEs were retained. Regions flanked by putative LTRs not showing similarity to known *Olea* retrotransposons were used as queries for Censor (www.girinst.org) to search for the most similar elements in the RepBase. Target site duplications (TSDs) were identified manually. Additional manipulations and alignments were performed in BioEdit 7.2.5 [93]. All names of olive LTR retrotransposons are reported after Barghini et al. [94].

To screen for the presence of cis-regulatory elements located at the promoter region that could be related with regulation of gene expression by auxins, a region comprising 1.5 Kb upstream the translation start site of both AOX1 gene sequences was considered for analysis. Promoter region of *OeAOX1a* (Oe6_s00216) and *OeAOX1d* (Oe6_s05781) was retrieved from the whole genome sequencing project (http://denovo.cnag.cat/genomes/olive/). The freely available New PLACE tool—A Database of Plant *Cis-*acting Regulatory DNA elements (https://sogo.dna.affrc.go.jp/cgi-bin/sogo.cgi?lang=en&pj=640&action=page&page=newplace, [95]) and PlantCARE (http://bioinformatics.psb.ugent.be/webtools/plantcare/html/, [96]) were used.

### 4.2. Transcript Expression of the AOX1-subfamily Members during AR on in Vitro Cultured Stem Segments

#### 4.2.1. Plant Material and in Vitro Rooting Experiments

The *in vitro* grown plantlets of cv. ”Galega vulgar” (clone 1441), described above, were used as initial explant source for the AR experiment. Rooting treatments and culture conditions were adapted from Macedo et al. [40]. In brief, stem segments (microcuttings) with four-to-five nodes were prepared from the upper part of in vitro-cultured plantlets and all leaves were removed with the exception of the upper four. The base (approx. 1.0 cm) of each microcutting was immersed in a sterile solution of 14.7 mM IBA (indole-3-butyric acid) [14] for 10 s. Subsequently, the microcuttings were aseptically inoculated in 500 mL glass flasks containing 75 mL semi-solid olive culture medium (OM), without plant growth regulators and supplemented with 7 g/L commercial agar-agar, 30 g/L d-mannitol, and 2 g/L activated charcoal [97]. Medium pH was adjusted to 5.8 prior autoclaving (20 min at 121 °C, 1 kg/cm^2^). Twenty microcuttings were inoculated per flask, and four flasks were used per time point (4 and 8 h and 1, 2, 4, 6, 8, 10, 14, 18, 22, 26, and 30 days). All cultures were kept in a plant growth chamber at 24 °C/21 °C (±1 °C) day/night temperatures, with a 15 h photoperiod, under cool-white fluorescent light at a photosynthetically active radiation (PAR) level of 36 μmol/m^2^ s^−2^ at culture height. During the in vitro rooting experiment, the segments from the basal portion (approx. 1 cm from the base) of the microcuttings were collected from each flask at the time points mentioned above. Basal segments from microcuttings prepared as described above, but not immersed in IBA and not inoculated in OM medium, were also collected and these corresponded to the time point 0 h (control group). All samples were flash frozen in liquid nitrogen and stored at −80 °C for subsequent analyses.

#### 4.2.2. RNA Isolation and First-Strand cDNA Synthesis

Total RNA was isolated with the Maxwell 16 LEV simplyRNA purification kit (Promega, Madison, WI, USA) on the Maxwell 16 Instrument (Promega, Madison, WI, USA) according to the supplier’s instructions, and eluted in 50 μL volume of RNase-free water. The concentration of total RNA was determined with the NanoDrop-2000C spectrophotometer (Thermo Scientific, Wilmington, DE, USA), and the total RNA integrity was analyzed by agarose gel electrophoresis through visualization of the two ribosomal subunits in a Gene Flash Bio Imaging system (Syngene, Cambridge, UK). GoScript Reverse Transcription System (Promega, Madison, WI, USA) was used to synthesize complementary DNA (cDNA) from RNA samples (using 1 μg of total RNA), according to the manufacturer’s instructions.

#### 4.2.3. Quantitative Real-Time PCR

Real-time PCR was performed in the Applied Biosystems 7500 Real-Time PCR System (Applied Biosystems, Foster City, CA, USA). Real-time PCR reactions were carried out using 1X Maxima SYBR Green qPCR Master Mix, 10 ng of cDNA, and the primers for each target gene and the corresponding transcript variants, as well as for the reference genes used here, as shown in Table S2, in a total volume of 18 μL. Primers for the *OeAOX1a* and *OeAOX1d* genes were designed to a common region in the exon 4 in order to detect and amplify all set of distinct transcripts variants. Primers to detect and amplify transcript variants for each gene were designed for a specific region for each variant (see primers localization in Figure 2 (for *OeAOX1a*) and Figure S6 (for *OeAOX1d*)). Primers were designed based on the *OeAOX1a* and *OeAOX1d* sequences deposited in the NCBI with the Primer Express v3.0 (Applied Biosystems, Foster City, CA, USA), using the default properties given by the software. Regarding the reference genes, actin (*OeACT*) and the elongation factor 1a (*OeEF1a*) were selected in a previous analysis as the most stable genes for this experimental system, following an analysis described by Velada et al. [27]. All primer pairs were checked for their probability to form dimers and secondary structures using the primer test tool of the software. The reactions were performed using the following thermal profile: 10 min at 95 °C, and 40 cycles of 15 s at 95 °C and 60 s at 60 °C. No-template controls (NTCs) were used to assess contaminations and primer dimers formation. A standard curve was performed using an undiluted pool containing all cDNA samples and three four-fold serial dilutions, with a total of four points. All samples were run in duplicate. Melting curve analysis was done to ensure amplification of the specific amplicon. Quantification cycle (Cq) values were acquired for each sample with the Applied Biosystems 7500 software (Applied Biosystems, Foster City, CA, USA).

#### 4.2.4. Expression Analysis of Transcripts

For expression levels normalization of the transcripts under study, *C*q values were converted into relative quantities (RQ) by the delta-*C*t method described by Vandesompele and co-authors [98]. The normalization factor was determined by the GeNorm algorithm [98] and corresponds to the geometric mean between the RQ of the selected reference genes for each sample. For each gene of interest, calculating the ratio between the RQ for each sample and the corresponding normalization factor, a normalized gene expression value was obtained. The graphs show the mean ± standard deviation of four biological replicates, and the bars represent the fold-change related to the control group (0 h), which was set to 1. Statistical significances (*p* ≤ 0.05, *p* ≤ 0.01, and *p* ≤ 0.001) between means were determined by the t-test method using the IBM^®^ SPSS^®^ Statistics version 22.0 (SPSS Inc., Armonk, NY, USA).

## Figures and Tables

**Table 1:** Information regarding the full length elements identified in the upstream and downstream region of *OeAOX1* genes in cv. ”Farga”. For visualization of elements position within genomic sequence see Figures S7 and S8.

Element	Length (bp)	LTR Length	TSD	Position Relative to Start Codon
*OeAOX1a*				
isolate 84856_A retrotransposon gypsy-type [KM577525]	13,288	1791 (left) 1752 (right)	GAAAG	−18,801/−5513
isolate 95401_B retrotransposon gypsy-type [KM577546]	12,998	782 (left) 773 (right)	GTCAT	−27,411/−1125
*OeAOX1d*				
isolate 95401_B retrotransposon gypsy-type [KM577546]	12,948	767 (left) 773 (right)	CAATT	−16,375/−3427
isolate 70744_E retrotransposon copia-type [KM577454]	6023	750 (left) 750 (right)	TTATC	undetermined (downstream)
unknown, copia-type, similar to Copia-63_VV-I	4959	275 (left) 283 (right)	[A/G]TAGC	undetermined (downstream)

## Supplementary Materials

Supplementary materials can be found at http://www.mdpi.com/1422-0067/19/2/597/s1.

## Abbreviations

AOX	Alternative oxidase
AR	Adventitious rooting
IBA	indole-3-butyric acid
AR	adventitious rooting
SNP	single nucleotide polymorphism
QTL	Quantitative Trait Locus
UTR	Untranslated region
APA	alternative polyadenylation
mRNA	Messenger Ribonucleic acid
PAS	poly(a) site
gDNA	Genomic Deoxyribonucleic acid
NCBI	National Center for Biotechnology Information
TSA	transcriptome shotgun assembly
NJ	Neighbor-Joining
RACE	Rapid amplification of cDNA ends
RT-qPCR	Reverse transcription-quantitative real time polymerase chain reaction
ORF	Open reading frame
cDNA	complementary DNA
LTR	long terminal repeats
TSD	Target site duplications
PCR	polymerase chain reaction
mTP	mitochondrial transit peptides
snoRNAs	Small nucleolar RNAs
TEs	transposable elements
NMD	nonsense-mediated decay
RBPs	RNA-binding proteins
SHAM	salicylhydroxamic acid
ROS	Reactive oxygen species
ACT	actin
EF1a	elongation factor 1a
Cq	Quantification cycle
RQ	relative quantities
