# Supplementary material for: AOX1-Subfamily Gene Members in Olea europaea cv. “Galega Vulgar”—Gene Characterization and Expression of Transcripts during IBA-Induced in Vitro Adventitious Rooting
Source: Int J Mol Sci. 2018 Feb 17;19(2):597. doi: 10.3390/ijms19020597 (PMC5855819; doi:10.3390/ijms19020597)
Supplement: Supplementary file 1 [file ijms-19-00597-s001.pdf]

Supplementary Tables and Figures

Table S1: Primers sequences used for RACE-PCR and complete gene isolation.

Gene	Primers sequence (5′→3′)	Aim
OeAOX1a	GSP1: CTAAGAATGTGACAGGGGCGTGGTG	5′ end isolation
	GSP2: ACCAAACTCGACTCTGCGCGATGTA	3′ end isolation
	OeAOX1a_14Fw:CCGTGTTGCATTATAGATCTTGTTC<br>OeAOX1a_1420Rv: ACGCAAAGCGATTACTAGTTCA	Complete gene isolation
OeAOX1d	OeAOX1d_5end: 5′-GCATTTGCAAATACACCTTGG-3′	5′ end isolation*
	GSP2: CGAGGCACACCATCGTGACCTTAAC	3′ end isolation
	OeAOX1dFw: TTATTTTGTGTTTATTTCTTTCTA<br>OeAOX1dRev: ATCATTTATTGTCATCTATTATCG	Complete gene isolation
	OeAOX1d_24Fw: ATTATTTTGTGTTTATTTCTTTCT<br>OeAOX1d_1085Rev: CCTTTTGTCCTTCAGTGGT	Complete gene isolation

* primers obtained from a previous work [14].

Table S2. Primers used for RT-qPCR analysis.

Gene	Acc. number	Primers sequence 5′→3′	AS (bp)	[nM]	E (%)
OeAOX1a	-	Fw: CGGCTATTGCCATCGACTAT<br>Rv: GGGATTCCTTCAGTTCATGTC	150	300<br>300	101
*OeAOX1a*_transcript variant X1	MG208095	Fw: CGGCTATTGCCATCGACTAT<br>Rv: CATCAGAAATTGTGATCAGTGAAA	311	900<br>900	110
*OeAOX1a*_transcript variant X2	MF410314	Fw: CGGCTATTGCCATCGACTAT<br>Rv: AAATACGCAAAGCGATTACTAGT	379	300<br>300	88
OeAOX1d	-	Fw: CTCAAATTCCCCACCTACTTGTTT<br>Rv: AGCTGCCACCGTCTCTAGGA	75	300<br>300	98
*OeAOX1d*_transcript variant X1	MF410315	Fw: GGCACACCATCGTGACCTTA<br>Rv: GGATATTCCTTTAGCTCGTGTCCTT	72	300<br>300	98
*OeAOX1d*_transcript variant X2	JX912721	Fw: GGCACACCATCGTGACCTTA<br>Rv: CGTCGACAAGGAAAATGAAAATCT	65	300<br>300	91
OeActin	AF545569	Fw: TTGCTCTCGACTATGAACAGG<br>Rv: CTCTCGGCCCCAATAGTAATA	106	300<br>300	95
OeEF1a	AM946404	Fw: TTTTGAGGGTGACAACATGAT<br>Rv: CAGGGTTGGGCCCTTGTAC	64	300<br>300	100

Acc. number: accession number at the NCBI databases (National Center for Biotechnology Information, http://www.ncbi.nlm.nih.gov/); AS: amplicon size; [nM]: final concentration of each primer in the RT-qPCR reaction mix; E: PCR efficiency.

Table S3. Eudicot plant species used in the NJ analysis. .

O	F	Species	Accession number
Brassicales	*Brassicaceae*	*Arabidopsis lyrata*	AL1G33660<br>AL3G24680<br>AL3G24690<br>AL5G06730<br>AL0G08000<br>AL8G30860
		Arabidopsis thaliana	AT1G32350<br>AT3G22360<br>AT3G22370<br>AT3G27620<br>AT5G64210
		Boechera stricta	Bostr.19424s0421.1<br>Bostr.19424s0422.1<br>Bostr.3359s0124.1<br>Bostr.0568s0383.1
		Brassica rapa	Bra010153<br>Bra001865<br>Bra031351<br>Bra023835<br>Bra037768
		Capsella grandiflora	Cagra.1189s0011.1<br>Cagra.1189s0012.1<br>Cagra.5575s0007.1<br>Cagra.3957s0019.1<br>Cagra.0248s0102.1
		Capsella rubella	Carubv10014065m<br>Carubv10019557m<br>Carubv10009739m<br>Carubv10026681m
		Eutrema salsugineum	Thhalv10021013m<br>Thhalv10009342m<br>Thhalv10005697m
		Thellungiella parvula	TP3G20130<br>TP3G20140<br>TP2G27270
	C	*Carica papaya*	evm.model.supercontig_8.29<br>evm.model.supercontig_42.47
Curcubi	Curc	*Citrullus lanatus*	CL10G07420
		Cucumis melo	CM00136G00010
		Cucumis sativus	Cucsa.398150.1
Fabales	*Fabaceae*	*Glycine max*	GM04G14800<br>GM08G07690<br>GM08G07700<br>GM05G24455
		Lotus japonicus	LJ2G020780<br>LJ4G005280<br>LJ4G005290
		Medicago truncatula	MT5G026620<br>MT5G070680

O	F	Species	Accession number
			MT5G070870<br>MT5G070880
		Phaseolus vulgaris	Phvul.002G127100.1<br>Phvul.002G209100.1<br>Phvul.002G209200.1
Malvales	***Malvaceae***	*Gossypium raimondii*	Gorai.008G296600.1<br>Gorai.012G142200.1<br>Gorai.005G220400.1<br>Gorai.005G220500.1
		Theobroma cacao	TC03G031300<br>TC02G011670
Malpighiales	Linac	*Linum usitatissimum*	Lus10035670<br>Lus10005372<br>Lus10020523
	Salicaceae	*Populus trichocarpa*	PT03G09340<br>PT12G01430<br>PT12G01440<br>PT15G01960
		Salix purpurea	SapurV1A.1470s0080.1<br>SapurV1A.0346s0170.1<br>SapurV1A.0377s0140.1<br>SapurV1A.0377s0150.1<br>SapurV1A.3352s0030.1<br>SapurV1A.0894s0160.1
	Eu	*Manihot esculenta*	ME10292G00060
		Ricinus communis	RC30063G00030
	H	*Hypericum perforatum*	EU330415.1<br>EU330413.1
Ranunc	Ranun	*Aquilegia coerulea Goldsmith*	Aquca_105_00003.1<br>Aquca_043_00024.1<br>Aquca_008_00134.1<br>Aquca_033_00110.1
Rosales	***Rosaceae***	*Fragaria vesca*	FV5G29310<br>FV5G21950
		Malus domestica	MD00G028680<br>MD00G081720<br>MD13G026910<br>MD16G016620
		Prunus persica	Prupe.5G018700.1<br>Prupe.1G061800.1<br>Prupe.1G061900.1<br>Prupe.1G061400.1
Sapindales	***Rutaceae***	*Citrus clementina*	Ciclev10001766m<br>Ciclev10003687m<br>Ciclev10028835m
		Citrus sinensis	orange1.1g037339m<br>orange1.1g019765m<br>orange1.1g020532m
Solan	***Solan***	*Solanum lycopersicum*	Solyc08g005550<br>Solyc08g075540<br>Solyc08g075550

O	F	Species	Accession number
			Solyc01g105220
		Solanum tuberosum	PGSC0003DMT400019708<br>PGSC0003DMT400019707<br>PGSC0003DMT400047562<br>PGSC0003DMG400012558
Amborellales	*Amborellaceae*	*Amborella trichopoda*	ATR_00048G01570<br>evm_27.model.AmTr_v1.0_scaffold00048.158<br>ATR_00038G01180<br>evm_27.model.AmTr_v1.0_scaffold00038.119
Caryophy	Amaranta	*Beta vulgaris*	BV5G19180<br>BV9G03180
		Amaranthus hypochondriacus	AHYPO_008129-RA<br>AHYPO_002393-RA<br>AHYPO_002394-RA
Saxifragales	***Crassulaceae***	*Kalanchoe laxiflora*	Kalax.0453s0008.1<br>Kalax.1476s0004.1<br>Kalax.0496s0017.1<br>Kalax.0907s0011.1<br>Kalax.0414s0014.1
		Kalanchoe fedtschenkoi	Kaladp0418s0013.1<br>Kaladp0067s0033.1<br>Kaladp0062s0189.1
Lamiales	Phry	*Mimulus guttatus*	Migut.J01127.1<br>Migut.E01358.1<br>Migut.N01067.1
	Olea	*Olea europaea*	Galega vulgar_MF410314*<br>Galega vulgar_MF410315*<br>Galega vulgar_JX912721*<br>Galega vulgar_JX912722<br>Dolce Agogia_KM514920<br>Leccino_KM514918<br>Dolce Agogia_KM514921<br>Leccino_KM514919<br>Leccino_GCJV01040584<br>Picual_GBKW01105538<br>Farga_FKYM01004812<br>Farga_FKYM01030627<br>Farga_FKYM01003481
Myrt	Myrt	*Eucalyptus grandis*	Eucgr.E01214.1<br>Eucgr.E01213.1<br>Eucgr.I02663.1
Vita	Vit	*Vitis vinifera*	VV02G09030<br>VV02G09050<br>VV00G00110

O: order; Caryophy*: Caryophyllales*; Curcubi*:Curcubitales*; Lam*: Lamiales*; Mytr*: Myrtales*; Ranunc*: Ranunculales*; Vita*: Vitales;* F: family; Amaranta: *Amarantaceae*; C: *Caricaceae*; Curc: *Curcubitaceae*; E: *Euphorbiaceae*; H: *Hypericaceae*; Lina:

Linaceae; Myr: *Myrtaceae*; Olea*: Oleaceae*; Phry*: Phrymaceae*; Ranun: *Ranunculaceae*; Vit: *Vitaceae*.; In grey the AOX2 sequences. *olive sequences characterized in the present research.

Table S4. Monocot plant species used in the NJ analysis.

In grey the AOX2 sequence.

Species	Accession number
Brachypodium distachyon	BD3G52505<br>BD5G20540<br>BD5G20547<br>BD5G20557
Brachypodium stacei	Brast04G094100.1<br>Brast09G194700.1<br>Brast09G194600.1<br>Brast09G194800.1
Hordeum vulgare	CAJW010038523<br>CAJW011587016<br>CAJW010099492
Musa acuminata	GSMUA_Achr5G03810_001<br>GSMUA_Achr6G01170_001<br>GSMUA_Achr6G01300_001<br>GSMUA_Achr1G27800_001
Oropetium thomaeum	Oropetium_20150105_19337A<br>Oropetium Seq1_20150105_01586A<br>Oropetium Seq2_20150105_01586A<br>Oropetium Seq3_20150105_01586A
Oryza brachyantha	OB02G22630<br>OB02G36280<br>OB04G30980<br>OB04G30990
Oryza glaberrima	ORGLA02G0249500<br>ORGLA04G0206000<br>ORGLA04G0206100
Oryza sativa	BGIOSGA008063<br>BGIOSGA005788<br>BGIOSGA014421<br>BGIOSGA014422
Panicum hallii	Pahal.G02176.1<br>Pahal.G02177.1<br>Pahal.G02175.1<br>Pahal.A03053.1
Panicum virgatum	Pavir.Ab01160.1<br>Pavir.Aa00784.1<br>Pavir.Ga00730.1<br>Pavir.Gb00786.1<br>Pavir.Ga00729.1<br>Pavir.Ga00625.1<br>Pavir.Ab02811.1<br>Pavir.Gb00785.1

	Pavir.Gb00789.1
Setaria italica	Seita.7G223800.1<br>Seita.7G223900.1<br>Seita.7G223700.1<br>Seita.1G286500.1
Setaria viridis	Sevir.7G235500.1<br>Sevir.7G235600.1<br>Sevir.7G235400.1<br>Sevir.1G291700.1
Spirodela polyrhiza	Spipo20G0025000<br>Spipo5G0073900<br>Spipo11G0008300
Sorghum bicolor	SB04G030820<br>SB06G027410<br>SB06G027420<br>SB06G027430
Zea mays	ZM02G05480<br>ZM02G05490<br>ZM02G05500<br>ZM05G37570

Table S5. Information regarding the cis-elements extracted from the 1.5 kbp upstream region of both OeAOX1 genes using the PlantCARE and New Place softwares.

	ID motif	Sequence	Locus (Strand)	Source	Specific function	References
OeAOX1a	NTBBF1ARROLB	ACTTTA	266 (-)	PLACE_S000273	Required for tissue-specific expression and auxin induction	Baumann et al. 1999
	ARFAT	TGTCTC	305 (+)	PLACE_S000270	ARF (auxin response factor) binding site found in the promoters of primary/early auxin response genes of Arabidopsis thaliana	Inukai et al. 2005, Nemhauser al. 2004, Goda et al. 2004, Hagen and Guilfoyle 2002
	SURECOREATSULTR11	GAGAC	306 (-)	PLACE_S000499	contains auxin response factor (ARF) binding sequence (see above ARFAT)	Maruyama et al. 2005
	BO~TGA-element	AACGAC	890 (-)	PlantCARE	auxin-responsive element	Pastuglia et al. 1997
OeAOX1d	ASF1MOTIFCAMV	TGACG	164 (-)	PLACE_S000024	TGACG motifs are found in many promoters and are involved in transcriptional activation of several genes by auxin and/or salicylic acid	Despres et al. 2003, Klinedinst et al. 2000
	NTBBF1ARROLB	ACTTTA	668 (-)<br>1109 (+)	PLACE_S000273	Required for tissue-specific expression and auxin induction	Baumann et al. 1999
	D4GMAUX28	TAGTGCTGT	1383 (-)	PLACE_S000331	DNase I protected sequence found in the *Glycine max* auxin responsive gene, Aux28, promoter	Nagao et al. 1993

Baumann K, De Paolis A, Costantino P, Gualberti G (1999). The DNA binding site of the Dof protein NtBBF1 is essential for tissue-specific and auxin-regulated expression of the rolB oncogene in plants. Plant Cell 11:323-333

Inukai Y, Sakamoto T, Ueguchi-Tanaka M, Shibata Y, Gomi K, Umemura I, Hasegawa Y, Ashikari M, Kitano H, Matsuoka M.(2005). Crown rootless1, which is essential for Crown root formation in rice, is a target of an AUXIN RESPONSE FACTOR in auxin signaling. Plant Cell 17: 1387-1396

Nemhauser JL, Mockler TC, Chory J. (2004). Interdependency of brassinosteroid and auxin signaling in Arabidopsis. PLoS Biol. 2(9):E258

Goda H, Sawa S, Asami T, Fujioka S, Shimada Y, Yoshida S.(2004). Comprehensive comparison of auxin-regulated and brassinosteroid-regulated genes in Arabidopsis. Plant Physiol. 134: 1555-1573

Hagen G, Guilfoyle T (2002). Auxin-responsive gene expression: genes, promoters and regulatory factors. Plant Mol Biol. 49 :373-385

Maruyama-Nakashita A, Nakamura Y, Watanabe-Takahashi A, Inoue E, RA Yamaya T, Takahashi H. (2005). Identification of a novel cis-acting element conferring sulfur deficiency response in Arabidopsis roots. Plant J. 42: 305-314

M Pastuglia, D Roby, C Dumas, J M Cock (1997). Rapid induction by wounding and bacterial infection of an S gene family receptor-like kinase gene in Brassica oleracea. Plant Cell. 1997 Jan; 9(1): 49–60.

Despres C, Chubak C, Rochon A, Clark R, Bethune T, Desveaux D, RA Fobert PR. (2003). The Arabidopsis NPR1 disease resistance protein is a novel cofactor that confers redox regulation of DNA binding activity to the basic domain/leucine zipper transcription factor TGA1. Plant Cell 15: 2181-2191

Klinedinst S, Pascuzzi P, Redman J, Desai M, Arias J. (2000). A xenobiotic-stress-activated transcription factor and its cognate target genes are preferentially expressed in root tip meristems. Plant Mol Biol 42: 679-688

Baumann K, De Paolis A, Costantino P, Gualberti G (1999). The DNA binding site of the Dof protein NtBBF1 is essential for tissue-specific and auxin-regulated expression of the rolB oncogene in plants. Plant Cell 11:323-333

Nagao RT, Goekjian VH, Hong JC, Key JL (1993). Identification of protein-binding DNA sequences in an auxin-regulated gene of soybean. Plant Mol Biol 21: 1147-1162

```
GAATTTCTCTACCATTTTCATTGATCCTATTTCTTTGATTATAATCTTCTGAAGAACAATGATGATAAAA 140
                                                            M  M  I  K

AGCACGACCAGGGTAGCACGGGCCGTGTTATGCCACATGGGCCCACGTTATTTTTCGACAACCGCCTTGC 210
 S  T  T  R  V  A  R  A  V  L  C  H  M  G  P  R  Y  F  S  T  T  A  L

ATGGCTGTGTAGCAAGTGATGTGCAAGCAATTAGGGGTGTTTTTGGCGGCACCACCACTTTCTTCCATGG 280
H  G  C  V  A  S  D  V  Q  A  I  R  G  V  F  G  G  T  T  T  F  F  H  G

CAATCTTGCCCAGAGTTCTGAAAAGGTGTTGGTAGGGTGCATGAGGTTGCTGGCGGTTGGTGGTGCGCGT 350
  N  L  A  Q  S  S  E  K  V  L  V  G  C  M  R  L  L  A  V  G  G  A  R

AAGGCGAGCACTTTGGCCTTGGGTGACAAACAGCAGGAGGAAGAGAAGAAGGTACAAGGAGGAGAAACTG 420
 K  A  S  T  L  A  L  G  D  K  Q  Q  E  E  E  K  K  V  Q  G  G  E  T

GTGGTGCCGCCGCTGCCGGTGGGGGCAATAATAACAAGGGAATAGTGAGTTATTGGGGCGTGGAGCCTGC 490
G  G  A  A  A  A  G  G  G  N  N  N  K  G  I  V  S  Y  W  G  V  E  P  A
                                                 ▼
CAAGATTACTAAAGAGGATGGCTCTGAATGGAGGTGGAACTGCTTTAAGCCATGGGAGACCTACAAGGCT 560
  K  I  T  K  E  D  G  S  E  W  R  W  N  C  F  K  P  W  E  T  Y  K  A

GATCTGTCTATAGATCTGAAGAAACACCATGCCCCTGTCACATTCTTAGACAAGGTGGCATATTGGACCG 630
 D  L  S  I  D  L  K  K  H  H  A  P  V  T  F  L  D  K  V  A  Y  W  T
                                      ▼
TCAAGTCTCTCAGATTTCCTACAGATATATTCTTTCAGAGGCGGTATGGATGTCGTGCTATGATGCTGGA 700
V  K  S  L  R  F  P  T  D  I  F  F  Q  R  R  Y  G  C  R  A  M  M  L  E

AACTGTGGCCGCTGTGCCTGGCATGGTTGGAGGGATGCTTCTGCACTGCAAGTCACTGAGGCGATTCGAG 770
  T  V  A  A  V  P  G  M  V  G  G  M  L  L  H  C  K  S  L  R  R  F  E

CACAGTGGTGGTTGGATCAAAGCATTGTTAGAAGAAGCCGAAAATGAAAGAATGCACCTCATGACATTCA 840
 H  S  G  G  W  I  K  A  L  L  E  E  A  E  N  E  R  M  H  L  M  T  F

TGGAAGTTTCCCAGCCCAGATGGTACGAACGCGCTCTTGTATTCACTGTGCAGGGCGTATTTTTCAATGC 910
M  E  V  S  Q  P  R  W  Y  E  R  A  L  V  F  T  V  Q  G  V  F  F  N  A

ATACTTCTTGACCTATCTCGTTTCCCCGAAATTGGCGCATCGGGTTGTGGGGTATTTGGAAGAGGAGGCG 980
  Y  F  L  T  Y  L  V  S  P  K  L  A  H  R  V  V  G  Y  L  E  E  E  A

ATCCACTCGTACACCGAGTTCTTGAAAGAGTTGGACAAGGGCACTATTGAGAATGTTCCTGCTCCGGCTA 1050
 I  H  S  Y  T  E  F  L  K  E  L  D  K  G  T  I  E  N  V  P  A  P  A

TTGCCATCGACTATTGGCGTATGCCACCAAACTCGACTCTGCGCGATGTAGTCATGGTAGTTAGAGCTGA 1120
I  A  I  D  Y  W  R  M  P  P  N  S  T  L  R  D  V  V  M  V  V  R  A  D
                                     ▼
CGAAGCTCACCACCGTGATGTTAACCATTTTGCATCGGACATTCATTATCAGGGACATGAACTGAAGGAA 1190
  E  A  H  H  R  D  V  N  H  F  A  S  D  I  H  Y  Q  G  H  E  L  K  E

TCCCCAGCTCCACTTGGATATCACTGAATGGTCTGAAGAACTATACTGGAGAAGTATATTTAACCAATAT 1260
 S  P  A  P  L  G  Y  H  *

CTATAATATCGAATAAGATATACTAATTTGTTAAATTTTCCTTTTCTTGAGATTGATTTCATTGTATGTT 1330
TTTCACTGATCACATCATAATAAGTGTTGAATGTCTGTTATTTTACGTTTTTGGTTGAGAAATATCATGA 1400
ACTAGTAATCGCTTTGCGTATTTATATTTCTTTTAAGGTATTTACTAAAAAAAAAAAAAAAA 1462
```

Figure S1. Nucleotide and deduced amino acid sequences of cDNA encoding *AOX1a* of *Olea europaea* L. cv. ‘Galega vulgar’; the sequence corresponds to the first transcript variant (*OeAOX1a_transcript variant X1*, acc. no. MF410314). Since no other ATG codons was found in the beginning of the resultant ORFs it was considered that the selected ones represent the correct initiation of translation. ▼ indicates the position of the three introns, * indicates stop codon.

```
ATGATGATAAAAAGCACGACCAGGGTAGCACGGGCCGTGTTATGCCACATGGGCCCACGTTATTTTTCGA 70
 M  M  I  K  S  T  T  R  V  A  R  A  V  L  C  H  M  G  P  R  Y  F  S

CAACCGCCTTGCATGGCTGTGTAGCAAGTGATGTGCAAGCAATTAGGGGTGTTTTTGGCGGCACCACCAC 140
T  T  A  L  H  G  C  V  A  S  D  V  Q  A  I  R  G  V  F  G  G  T  T  T

TTTCTTCCATGGCAATCTTGCCCAGAGTTCTGAAAAGGTGTTGGTAGGGTGCATGAGGTTGCTGGCGGTT 210
  F  F  H  G  N  L  A  Q  S  S  E  K  V  L  V  G  C  M  R  L  L  A  V

GGTGGTGCGCGTAAGGCGAGCACTTTGGCCTTGGGTGACAAACAGCAGGAGGAAGAGAAGAAGGTACAAG 280
 G  G  A  R  K  A  S  T  L  A  L  G  D  K  Q  Q  E  E  E  K  K  V  Q

GAGGAGAAACTGGTGGTGCCGCCGCTGCCGGTGGGGGCAATAATAACAAGGGAATAGTGAGTTATTGGGG 350
G  G  E  T  G  G  A  A  A  A  G  G  G  N  N  N  K  G  I  V  S  Y  W  G
                                                             ▼
CGTGGAGCCTGCCAAGATTACTAAAGAGGATGGCTCTGAATGGAGGTGGAACTGCTTTAAGCCATGGGAG 420
  V  E  P  A  K  I  T  K  E  D  G  S  E  W  R  W  N  C  F  K  P  W  E

ACCTACAAGGCTGATCTGTCTATAGATCTGAAGAAACACCATGCCCCTGTCACATTCTTAGACAAGGTGG 490
 T  Y  K  A  D  L  S  I  D  L  K  K  H  H  A  P  V  T  F  L  D  K  V
                                                  ▼
CATATTGGACCGTCAAGTCTCTCAGATTTCCTACAGATATATTCTTTCAGAGGCGGTATGGATGTCGTGC 560
A  Y  W  T  V  K  S  L  R  F  P  T  D  I  F  F  Q  R  R  Y  G  C  R  A

TATGATGCTGGAAACTGTGGCCGCTGTACCTGGAATGGTCGGAGGGATGCTTCTGCACTGCAAGTCACTG 630
  M  M  L  E  T  V  A  A  V  P  G  M  V  G  G  M  L  L  H  C  K  S  L

AGGCAATTCGAGCACAGTGGTGGTTGGATCAAAGCATTGTTAGAAGAAGCCGAAAATGAAAGAATGCACC 700
 R  Q  F  E  H  S  G  G  W  I  K  A  L  L  E  E  A  E  N  E  R  M  H

TCATGACATTCATGGAAGTTTCCCAGCCCAGATGGTACGAACGCGCTCTTGTATTCACTGTGCAGGGCGT 770
L  M  T  F  M  E  V  S  Q  P  R  W  Y  E  R  A  L  V  F  T  V  Q  G  V

ATTTTTCAATGCCTACTTCTTGACCTATCTCGCTTCCCCGAAATTGGCTCATCGGATCGTGGGGTATTTG 840
  F  F  N  A  Y  F  L  T  Y  L  A  S  P  K  L  A  H  R  I  V  G  Y  L

GAAGAGGAGGCGATCCACTCGTACACCGAGTTCTTGAAAGAGTTGGACAAGGGCACTATTGAGAATGTTC 910
 E  E  E  A  I  H  S  Y  T  E  F  L  K  E  L  D  K  G  T  I  E  N  V

CTGCTCCGGCTATTGCCATCGACTATTGGCGTATGCCACCAAACTCGACTCTGCGCGATGTAGTCACGGT 980
P  A  P  A  I  A  I  D  Y  W  R  M  P  P  N  S  T  L  R  D  V  V  T  V
                                                 ▼
GGTTAGAGCTGACGAGGCTCATCACTGTGATGTTAACCATTTTGCATCGGACATTCATTATCAGGGACAT 1050
  V  R  A  D  E  A  H  H  C  D  V  N  H  F  A  S  D  I  H  Y  Q  G  H

GAACTGAAGGAAGCCCCGGCCCCAATTGGATATCACTGAATGGTCTGAAGAACTATACCTGGAGAAGTAT 1120
 E  L  K  E  A  P  A  P  I  G  Y  H  *

ATTTAACCAATATCTATAATATTGAATAAGATATACTAATATGTTAAATTTTCCTTTTCTTAAGGTTGAT 1190
TTCACTGTACATTTTTCACTGATCACAATTTCTGATGGATAATAAAAAAAAAAAAAAAA 1249
```

Figure S2. Nucleotide and deduced amino acid sequences of cDNA encoding *AOX1a* of *Olea europaea* L. cv. 'Galega vulgar'; the sequence corresponds to the second transcript variant (*OeAOX1a_transcript variant X2*, acc. no. MG208095). Since no other ATG codons was found in the beginning of the resultant ORFs it was considered that the selected ones represent the correct initiation of translation. ▼ indicates the position of the three introns, * indicates stop codon.

```
ACACCAAAATCAAAGCTCAAAATACAATTCAAATTATTTTGTGTTTATTTCTTTCTATATTCAAAATCCC 70
GAATTCCAATGAGCCAACGTACAATTTCTAGTATGGTTTTTCGACAGATGCAGTCGAATTTTTCATCTTT 140
         M  S  Q  R  T  I  S  S  M  V  F  R  Q  M  Q  S  N  F  S  S  F

TAGTAGTTCGATGAATAATGTCTCCAAGAACTACCGACCCGCAATCACTCATATTTTTGAGGCAAGGTAC 210
  S  S  S  M  N  N  V  S  K  N  Y  R  P  A  I  T  H  I  F  E  A  R  Y

TATAGTAGTAACCTAGGTTCAAAAGGTAACAAAGAAGACGAGCCAGCACAAGCTGTGAAATTCGATTCCA 280
 Y  S  S  N  L  G  S  K  G  N  K  E  D  E  P  A  Q  A  V  K  F  D  S

ACTTTGAAAACGTCGATGGCCAAAATGGTAAGGCCGTCGTAAGCAGCTACTGGGGAGTACCTCCGTCGAG 350
N  F  E  N  V  D  G  Q  N  G  K  A  V  V  S  S  Y  W  G  V  P  P  S  R
                                                ▼
GGCGACCAAGGAGGATGGATCGCCCTGGCGATGGAATTGTTTTCGGCCATGGGAGACTTATAAAGCGGAC 420
  A  T  K  E  D  G  S  P  W  R  W  N  C  F  R  P  W  E  T  Y  K  A  D

ACTTCAATTGATGTGACAAAGCACCACAAGGCAACTACGTTCATGGACAAATTTGCCTATTGGACTGTTC 490
 T  S  I  D  V  T  K  H  H  K  A  T  T  F  M  D  K  F  A  Y  W  T  V
                                     ▼
AATCTCTCAAATTCACCTTAACCACTTTGCATCGAGACGCCACATGTGCCACGCTATGCTCCTAGAGAC 560
Q  S  L  K  F  P  T  Y  L  F  F  Q  R  R  H  M  C  H  A  M  L  L  E  T

GGTGGCAGCTGTCCCGGGCATGGTGGGGGGATGCTCCTACACTTAAAGTCGATCCGGCGGTTTGAACAC 630
  V  A  A  V  P  G  M  V  G  G  M  L  L  H  L  K  S  I  R  R  F  E  H

AGCGGTGGTTGGATCAAAGCCCTTCTCGAGGAAGCGGAAAATGAGAGAATGCATCTAATGACATTCTTAG 700
 S  G  G  W  I  K  A  L  L  E  E  A  E  N  E  R  M  H  L  M  T  F  L

AACTATCCCAACCGAAATGGTACCAGAGAGCCCTAGTATTTGCTGTCCAAGGTGTATTTGCAAATGCATA 770
E  L  S  Q  P  K  W  Y  Q  R  A  L  V  F  A  V  Q  G  V  F  A  N  A  Y

CTTTGTGTCCTATGTTGTGTCCCCAAAACTTGCTCATCGCATAGTAGGCTACCTTGAAGAAGAGGCAGTG 840
  F  V  S  Y  V  V  S  P  K  L  A  H  R  I  V  G  Y  L  E  E  E  A  V

AATTCATACACTGAATTTCTAATTGATTTGGAGAAGGGCCTTGTTGAAAATAGACCGGCGCCGGCAATCG 910
 N  S  Y  T  E  F  L  I  D  L  E  K  G  L  V  E  N  R  P  A  P  A  I

CCATTGATTACTGGCAGTTGCCGTCGGAATCAACGTTAAAAGATGTTGTCACGGTTATCAGGGCAGACGA 980
A  I  D  Y  W  Q  L  P  S  E  S  T  L  K  D  V  V  T  V  I  R  A  D  E
                                    ▼
GGCACACCATCGTGACCTTAACCACTTTGCATCGGACATTCAATGTGAAGGACACGAGCTAAAGGAATAT 1050
  A  H  H  R  D  L  N  H  F  A  S  D  I  Q  C  E  G  H  E  L  K  E  Y

CCTGCCCCGCTGGGATACCACTGAAGGACAAAAGGTTATTGAATAGAAAAAATTGCATACATATTTATTT 1120
 P  A  P  L  G  Y  H  *

TATTATTTTGAAAAGTAATTTCTGTAATTTAGAATAGAATAAATGCATATATATTTATTTTATTATTTTG 1190
AAAAGTAATTTCTGTAATTTCTATTTTTGTACTAAAATAATATTATTTCTCCTTTCATAAAAAAAAAAAA 1260
AAAAAAAAAAAAAAAAA 1277
```

Figure S3. Nucleotide and deduced amino acid sequences of cDNA encoding *AOX1d* of *Olea europaea* L. cv. 'Galega vulgar'; the sequence corresponds to the first transcript variant (*OeAOX1d_transcript variant X1,* acc. no. MF410315). Since no other ATG codons was found in the beginning of the resultant ORFs it was considered that the selected ones represent the correct initiation of translation. ▼ indicates the position of the three introns, * indicates stop codon.

```
ACACCAAAATCAAAGCTCAAAATACAATTCAAATTATTTTGTGTTTATTTCTTTCTATATTCAAAATCCC 70
GAATTCCAATGAGCCAACGTACAATTTCTAGTATGGTTTTTCGACAGATGCAGTCGAATTTTTCATCTTT 140
         M  S  Q  R  T  I  S  S  M  V  F  R  Q  M  Q  S  N  F  S  S  F

TAGTAGTTCGATGAATAATGTCTCCAAGAACTACCGACCCGCAATCACTCATATTTTTGAGGCAAGGTAC 210
 S  S  S  M  N  N  V  S  K  N  Y  R  P  A  I  T  H  I  F  E  A  R  Y

TATAGTAGTAACCTAGGTTCAAAAGGTAACAAAGAAGACGAGCCAGCACAAGCTGTGAAATTCGATTCCA 280
 Y  S  S  N  L  G  S  K  G  N  K  E  D  E  P  A  Q  A  V  K  F  D  S

ACTTTGAAAACGTCGATGGCCAAAATGGTAAGGCCGTCGTAAGCAGCTACTGGGGAGTACCTCCGTCGAG 350
N  F  E  N  V  D  G  Q  N  G  K  A  V  V  S  S  Y  W  G  V  P  P  S  R
                                                 ▼
GGCGACCAAGGAGGATGGATCGCCCTGGCGATGGAATTGTTTTCGGCCATGGGAGACTTATAAAGCGGAC 420
  A  T  K  E  D  G  S  P  W  R  W  N  C  F  R  P  W  E  T  Y  K  A  D

ACTTCAATTGATGTGACAAAGCACCACAAGGCAACTACGTTCATGGACAAATTTGCCTATTGGACTGTTC 490
 T  S  I  D  V  T  K  H  H  K  A  T  T  F  M  D  K  F  A  Y  W  T  V
                                      ▼
AATCTCTCAAATTCCCCACCTACTTGTTTTTTCAGAGACGCCACATGTGCCACGCTATGCTCCTAGAGAC 560
Q  S  L  K  F  P  T  Y  L  F  F  Q  R  R  H  M  C  H  A  M  L  L  E  T

GGTGGCAGCTGTCCCGGGCATGGTGGGGGGATGCTCCTACACTTAAAGTCGATCCGGCGGTTTGAACAC 630
  V  A  A  V  P  G  M  V  G  G  M  L  L  H  L  K  S  I  R  R  F  E  H

AGCGGTGGTTGGATCAAAGCCCTTCTCGAGGAAGCGGAAAATGAGAGAATGCATCTAATGACATTCTTAG 700
 S  G  G  W  I  K  A  L  L  E  E  A  E  N  E  R  M  H  L  M  T  F  L

AACTATCCCAACCGAAATGGTACCAGAGAGCCCTAGTATTTGCTGTCCAAGGTGTATTTGCAAATGCATA 770
E  L  S  Q  P  K  W  Y  Q  R  A  L  V  F  A  V  Q  G  V  F  A  N  A  Y

CTTTGTGTCCTATGTTGTGTCCCCAAAACTTGCTCATCGCATAGTAGGCTACCTTGAAGAAGAGGCAGTG 840
  F  V  S  Y  V  V  S  P  K  L  A  H  R  I  V  G  Y  L  E  E  E  A  V

AATTCATACACTGAATTTCTAATTGATTTGGAGAAGGGCCTTGTTGAAAATAGACCGGCGCCGGCAATCG 910
 N  S  Y  T  E  F  L  I  D  L  E  K  G  L  V  E  N  R  P  A  P  A  I

CCATTGATTACTGGCAGTTGCCGTCGGAATCAACGTTAAAAGATGTTGTCACGGTTATCAGGGCAGACGA 980
A  I  D  Y  W  Q  L  P  S  E  S  T  L  K  D  V  V  T  V  I  R  A  D  E

GGCACACCATCGTGACCTTAACCACTTTGCATCGGTAAGAAAGATTTTCATTTTCCTTTTCGACGAAAAT 1050
  A  H  H  R  D  L  N  H  F  A  S  V  R  K  I  F  I  F  L  F  D  E  N

ATTCTTAATACTTCAAACTTGTTAGAAGAGGTAATTGATCAATATGGTACCAAAATCGAGAAAGTCTTTA 1120
 I  L  N  T  S  N  L  L  E  E  V  I  D  Q  Y  G  T  K  I  E  K  V  F

TAATAAAAATTAATAAATAATATTCTTCTACGTAGTAGTTTGAATTTGAGAAATGCTACACTTACAAAAT 1190
I  I  K  I  N  K  *

AGACCCTTGCCAGATCTTTCACATGTGCGGACCCCTACGTGGGACCGACCCCTACAATGAGGGGTCGATA 1260
AAACATCTGTAGGAAATTGTTACGTACACAGAGCAATTTCCTTTGAATTTTTGATGAAAGATCGCACAAT 1330
TCCTAGATATTTTGTCAAATAAGAAATTTTCGATAATAGATGACAATAAATGATATCTAGTAGTGGATAA 1400
ATAAAATAACTTATTTTTCTAATAATTAGGTCGTACAAAAATTTCTTTATTATTCAAGTGAATGTCTGGT 1470
CTAAGTACTAAAAGGGATTTTTGACACTATAATATATAAAAGTTCAATTTTTATTAATAATCTATAATTC 1540
GTTTAATGTCCTAATAAGGAGTAATAATTTGTGAAATGAAACAAAAAAAAAAAAAAA 1597
```

Figure S4. Nucleotide and deduced amino acid sequences of cDNA encoding *AOX1d* of *Olea europaea* L. cv. 'Galega vulgar'; the sequence corresponds to the second transcript variant (*OeAOX1d_transcript variant X2,* acc. no. JX912721). Since no other ATG codons was found in the beginning of the resultant ORFs it was considered that the selected ones represent the correct initiation of translation. ▼ indicates the position of the three introns, * indicates stop codon.

```
ATGATGATAAAAAGCACGACCAGGGTAGCACGGGCCGTGTTATGCCACATGGGCCCACGTTATTTTTCGACAACCGCCTT
GCATGGCTGTGTAGCAAGTGATGTGCAAGCAATTAGGGGTGTTTTTGGCGGCACCACCACTTTCTTCCATGGCAATCTTG
CCCAGAGTTCTGAAAAGGTGTTGGTAGGGTGCATGAGGTTGCTGGCGGTTGGTGGTGCGCGTAAGGCGAGCACTTTGGCC
TTGGGTGACAAACAGCAGGAGGAAGAGAAGAAGGTACAAGGAGGAGAAACTGGTGGTGCCGCCGCTGCCGGTGGGGGCAA
TAATAACAAGGGAATAGTGAGTTATTGGGGCGTGGAGCCTGCCAAGATTACTAAAGAGGATGGCTCTGAATGGAGGTGGA
ACTGCTTTAAGGTATGTAGGAAAAAAATTAAGAACTTCTATATTATTATTTTGGTTCATATTCAAATTTTATGAATTAAT
TTGATTTGGCCTTTTGAATTTAATGATTTATGAAGCCATGGGAGACCTACAAGGCTGATCTGTCTATAGATCTGAAGAAA
CACCATGCCCCTGTCACATTCTTAGACAAGGTGGCATATTGGACCGTCAAGTCTCTCAGATTTCCTACAGATATATTCTT
TCAGGTATGTTATTATATTCTCTAGCTTGGTTTAATAAAAGAACAGTTTTTTTTTTTTTTCAGATTATGATAATTTGTT
GAAATGAACAGTTGAAAAGTTGATAATAATTATGAGTTTCATGCGTGTTGAAAATGAATTCGACGAAAAAAAAAAAAAAGA
TTTTCAATAAATAGTAGGGTTAATGTTCTTGGATTCAAGTCTTTTAGTGAAAAAAAATGAATTTCTATATTCTTTAGGCT
TTCCTTCCATCCTGTTTGGTTTAGCTAATTTTAGTCAAAACCTGGTAAAAATGGAAGTTAAGAGTCCATTTGGTACACAT
TAATGTAAAAATGAGAATGTAAAATATAGTCGTTATGGACCTCACACAAGAGATAGAAATTCAAATTTGGTGGCTCGATG
TGATGGATAATAATCATATTTTTCATTTTAATGAAGAAAAAATGGGAAACAAAAGGGAGCTAAAATTTAATAATTAATTT
TAACCATGAAATATGTGATCCATGACTCTTATCACTTTTCAATGATATATTTCATCAACTTTTATAAAAATTTATTGAAA
ATCTAATTTTTAATGCGAAAACTGAAACCAATCTCTATTTTGAACGTATGTAGTTACAAAAAGTTAAAAAGAAAGTAGAA
GGGACAAATTAAAATCTTTGAGAGTGTATGTAGTTTATTAGTTGAGTTTGGATGTCAACAAATCGAAAAGTGTGGCACAT
AGCCAACAATTATCAGAAAAAATGCTTAGCTGTTGTTCTTTCTATTAGATGGAAAGATACAGGGGGTTAGATGTTGTATT
GCATTTATGGTTTCTTTGGTGGATTAAACTTTATATTAGTCAAACCTATTAATCAAGCAAAAGTGGATACTTGTTTGTTG
GATTATTGTGTCTCGTGCAATAGTGCATACCAAACAACGCATTTAATTACTTTATCCTGTTACAGAGGCGGTATGGATGT
CGTGCTATGATGCTGGAAACTGTGGCCGCTGTGCCTGGCATGGTTGGAGGGATGCTTCTGCACTGCAAGTCACTGAGGCG
ATTCGAGCACAGTGGTGGTTGGATCAAAGCATTGTTAGAAGAAGCCGAAAATGAAAGAATGCACCTCATGACATTCATGG
AAGTTTCCCAGCCCAGATGGTACGAACGCGCTCTTGTATTCACTGTGCAGGGCGTATTTTTCAATGCATACTTCTTGACC
TATCTCGTTTCCCCGAAATTGGCGCATCGGGTTGTGGGGTATTTGGAAGAGGAGGCGATCCACTCGTACACCGAGTTCTT
GAAAGAGTTGGACAAGGGCACTATTGAGAATGTTCCTGCTCCGGCTATTGCCATCGACTATTGGCGTATGCCACCAAACT
CGACTCTGCGCGATGTAGTCATGGTAGTTAGAGCTGACGAAGCTCACCACCGTGATGTTAACCATTTTGCATCGGTACGT
GCATAAAACTTCATATGGTTTGATATTCAAAGCAATTTGATTTAGATGCTTAGGGTTTATTTGTAATGATTGCAGGACAT
TCATTATCAGGGACATGAACTGAAGGAATCCCCAGCTCCACTTGGATATCACTGA
```

Figure S5. Genomic sequence of *AOX1a* from *Olea euroaea* L. cv. 'Galega vulgar' (acc. no. MF410318) including the indication of the three exons with conserved sequence size (129, 489 and 57 bp, respectively) interrupted by three size variable introns.

```
ATGAGCCAACGTACAATTTCTAGTATGGTTTTCGACAGATGCAGTCGAATTTTTCATCTTTTAGTAGTTCGATGAATAA
TGTCTCCAAGAACTACCGACCCGCAATCACTCATATTTTTGAGGCAAGGTACTATAGTAGTAACCTAGGTTCAAAAGGTA
ACAAAGAAGACGAGCCAGCACAAGCTGTGAAATTCGATTCCAACTTTGAAAACGTCGATGGCCAAAATGGTAAGGCCGTC
GTAAGCAGCTACTGGGGAGTACCTCCGTCGAGGGCGACCAAGGAGGATGGATCGCCCTGGCGATGGAATTGTTTTCGGGT
ACGTACCCTAAAATTGTAAGAATTTTGCCATTTGGTGTTATGAAATAGAATAGAAAACACAATTTATGGTCAATGTTTAT
GATCAGTTTTTGAATTTGCATTTTTCCAGCCATGGGAGACTTATAAAGCGGACACTTCAATTGATGTGACAAAGCACCAC
AAGGCAACTACGTTCATGGACAAATTTGCCTATTGGACTGTTCAATCTCTCAAATTCCCCACCTACTTGTTTTTTCAGGT
TACACAATCATTTCCTGTTACCCATCTTAAAACTTGTCAATCATGTCCATTTTTGATAATAAGTTTCGAGTATCGTTCAT
ATTTAGTACGCAGTTTGGGCACTATACTATCGTCCAAACAAATTCCGTAATCTATAGAACGTCTTGGATATCGTCAACTA
TTTTTAATTCACTTATATTTCTAAATTCCTGACTGATAATTTCTTGTAGAGACGCCACATGTGCCACGCTATGCTCCTAG
AGACGGTGGCAGCTGTCCCGGGCATGGTGGGGGGATGCTCCTACACTTAAAGTCGATCCGGCGGTTTGAACACAGCGGT
GGTTGGATCAAAGCCCTTCTCGAGGAAGCGGAAAATGAGAGAATGCATCTAATGACATTCTTAGAACTATCCCAACCGAA
ATGGTACGAGAGAGCCCTAGTATTTGCTGTCCAAGGTGTATTTGCAAATGCCTATTTTGTGTCCTATGTTGTGTCCCCAA
AACTTGCTCATCGCATAGTAGGCTACCTTGAAGAAGAGGCAGTGAATTCATACACTGAATTTCTAATTGATTTGGAAAAG
GGCCTTGTTGAAAATAGACCGGCGCCGGCAATCGCCATTGATTACTGGCAGTTGCCGTCGGAATCGACATTAAAAGATGT
TGTCACGGTTATCAGGGCAGACGAGGCACACCATCGTGACCTTAACCACTTTGCATCGGTAAGAAAGATTTTCATTTTCC
TTGTCGACGAAAATATTTTTAATACTTCAAACCTGTTAGAAGAGATAATTGATCAATATGGTACCAAATTCGAGAAAGTC
TTTATAATAAAAATTAATAAATAATAATTCTTCTGTGTAGTAGTTTGAATTTTAGATGAAAGATCGTACAATTCCTAGAC
ATTTTGTAAAATAAGAAATTTTCGATAATAGATGACGATAAATGATATCTAGTAGCGGATAAATAAAATAACTTATTTTT
CTAATAATTAGGTTGTACAAAAATTTCTTTATTAAAGGTTGAACCCCAACCTTAAGGGTCCCCCACCGTCCTTGTTCTGA
AGTATCGTGAGGGAGGTAAATCGCAGGTTATGCCTCCAAGAGTACAGGGTGGAAGGCTGCTTGTACACTTGCACTTACCC
AAGAGCCAAATCCATATTTCAAGATTCGAACCCTTGATGCGACAGCCTCAAGTAAAACTGGATTTCTTTATTATTCAAGT
GAACATCTGGTCTAAGAACTAAAAGGGATTTTTGACAATACAATATACAAAATTTCACTTTTTATTAATAATCTATAATT
CGTTTAATGTCGTAATAAGGAGTAATAAATTGTGAAATGAAAAAGAAAAAAAGAAAAAACACAATCACTTAATTTTTTTA
TAATGACTTAATTTCCTTTAATATGCAGGACATTCAATGTGAAGGACACGAGCTAAAGGAATATCCTGCCCCGCTGGGAT
ACCACTGA
```

Figure S6. Genomic sequence of *AOX1d* from *Olea euroapea* L. cv. 'Galega vulgar' (acc. no. MF410319) including the indication of the exons/introns structure (exons coloured in green). The indication of the four exons structure with size conservation observed at the three last exons (129, 489 and 57 bp, respectively) known as the most typical *AOX* gene structure described in higher plants corresponds to transcript variant X1; the intron 3 region that is transcribed at transcript variant X2 instead of exon 4 is here indicated in red. Primers used in RT-qPCR analysis for *OeAOX1d* gene (designed to a common region) are indicated in yellow and for transcript variants X1 and X2 are indicated in blue.

I.

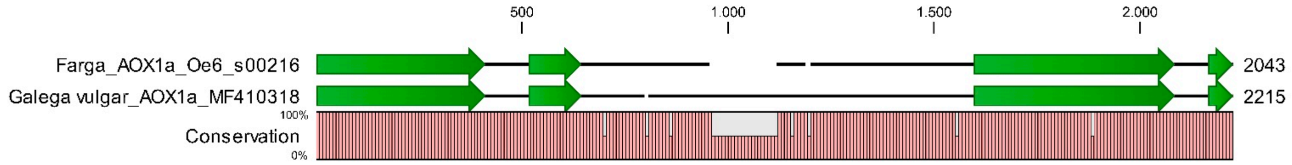

II.

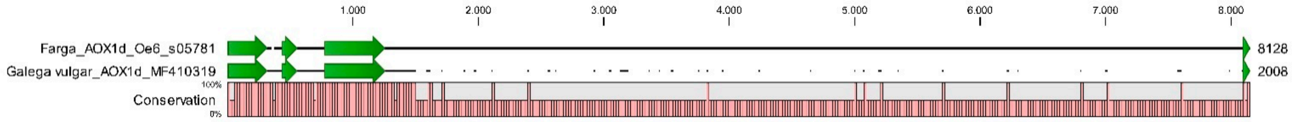

Figure S7. Comparison of *OeAOX* gene sequences isolated in cv. "Galega vulgar" with the sequences available from the olive whole genome sequencing databases in the cv. 'Farga'. I: *OeAOX1a* gene, II: *OeAOX1d* gene structure.

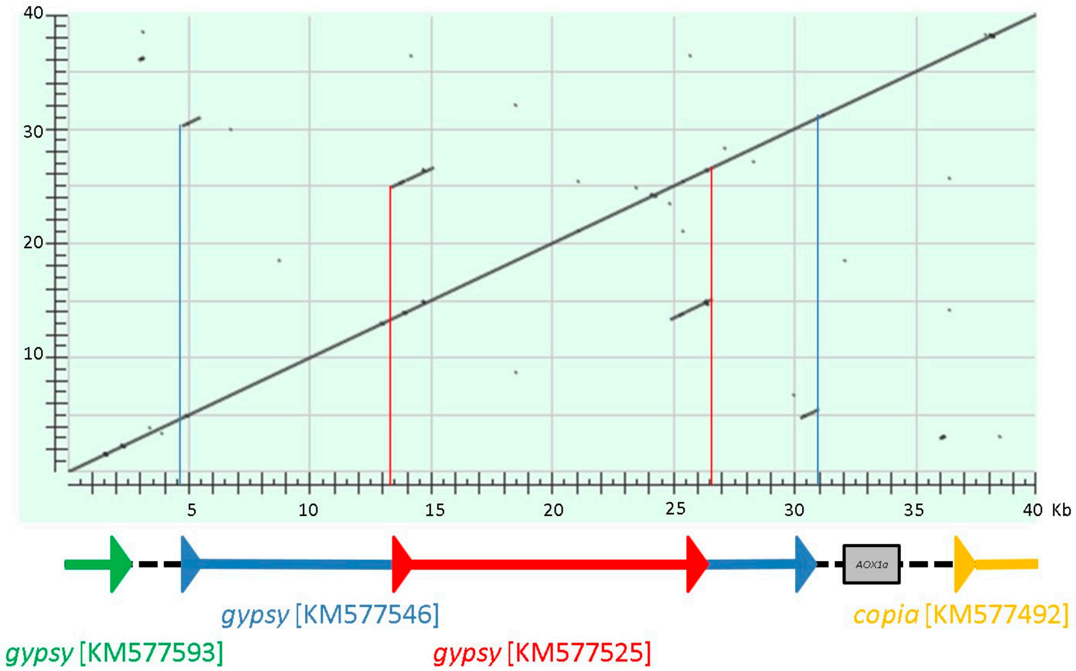

Figure S8. Location of the regulatory elements identified upstream and downstream the *OeAOX1a* gene in cv. 'Farga'.

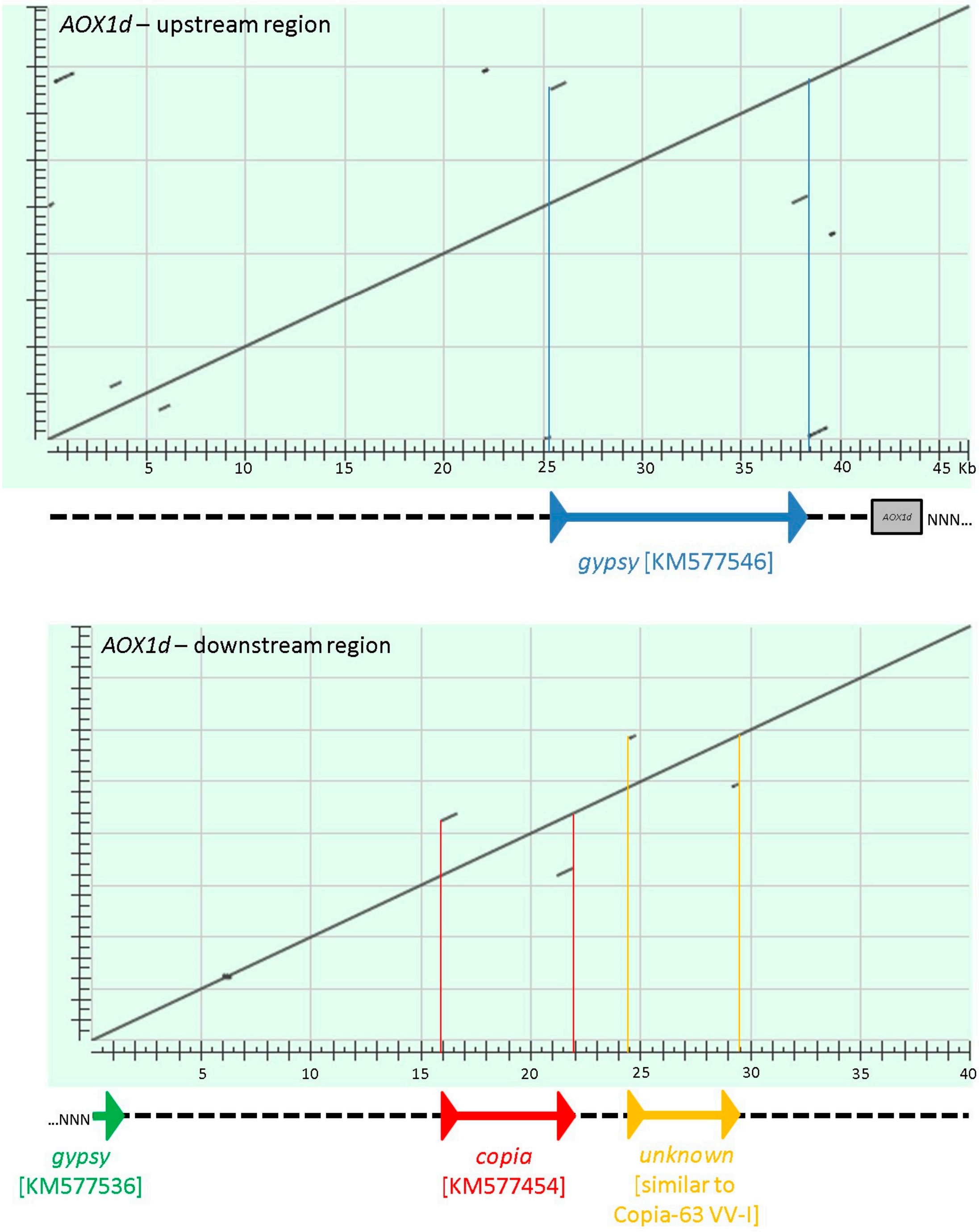

Figure S9. Location of the regulatory elements identified upstream and downstream the *OeAOX1d* gene in cv. 'Farga'.
